# Establishment of a Reverse Genetics System for Studying Human Bocavirus in Human Airway Epithelia

**DOI:** 10.1371/journal.ppat.1002899

**Published:** 2012-08-30

**Authors:** Qinfeng Huang, Xuefeng Deng, Ziying Yan, Fang Cheng, Yong Luo, Weiran Shen, Diana C. M. Lei-Butters, Aaron Yun Chen, Yi Li, Liang Tang, Maria Söderlund-Venermo, John F. Engelhardt, Jianming Qiu

**Affiliations:** 1 Department of Microbiology, Molecular Genetics and Immunology, University of Kansas Medical Center, Kansas City, Kansas, United States of America; 2 College of Life Sciences, Central China Normal University, Wuhan, China; 3 Department of Anatomy and Cell Biology, College of Medicine, University of Iowa, Iowa City, Iowa, United States of America; 4 Department of Molecular Biosciences, University of Kansas, Lawrence, Kansas, United States of America; 5 Haartman Institute, Department of Virology, University of Helsinki, Helsinki, Finland; King's College London School of Medicine, United Kingdom

## Abstract

Human bocavirus 1 (HBoV1) has been identified as one of the etiological agents of wheezing in young children with acute respiratory-tract infections. In this study, we have obtained the sequence of a full-length HBoV1 genome (including both termini) using viral DNA extracted from a nasopharyngeal aspirate of an infected patient, cloned the full-length HBoV1 genome, and demonstrated DNA replication, encapsidation of the ssDNA genome, and release of the HBoV1 virions from human embryonic kidney 293 cells. The HBoV1 virions generated from this cell line-based production system exhibits a typical icosahedral structure of approximately 26 nm in diameter, and is capable of productively infecting polarized primary human airway epithelia (HAE) from the apical surface. Infected HAE showed hallmarks of lung airway-tract injury, including disruption of the tight junction barrier, loss of cilia and epithelial cell hypertrophy. Notably, polarized HAE cultured from an immortalized airway epithelial cell line, CuFi-8 (originally derived from a cystic fibrosis patient), also supported productive infection of HBoV1. Thus, we have established a reverse genetics system and generated the first cell line-based culture system for the study of HBoV1 infection, which will significantly advance the study of HBoV1 replication and pathogenesis.

## Author Summary

Human bocavirus 1 (HBoV1) has been identified as one of the etiological agents of wheezing in young children with acute respiratory-tract infections. HBoV1 productively infects polarized primary human airway epithelia. However, no cell lines permissive to HBoV1 infection have yet been established. More importantly, the sequences at both ends of the HBoV1 genome have remained unknown. We have resolved both of these issues in this study. We have sequenced a full-length HBoV1 genome and cloned it into a plasmid. We further demonstrated that this HBoV1 plasmid replicated and produced viruses in human embryonic kidney 293 cells. Infection of these HBoV1 progeny virions produced obvious cytopathogenic effects in polarized human airway epithelia, which were represented by disruption of the epithelial barrier. Moreover, we identified an airway epithelial cell line supporting HBoV1 infection, when it was polarized. This is the first study to obtain the full-length HBoV1 genome, to demonstrate pathogenesis of HBoV1 infection in human airway epithelia, and to identify the first cell line to support productive HBoV1 infection.

## Introduction

Human bocavirus 1 (HBoV1) was initially identified in 2005, in nasopharyngeal aspirates of patients with acute respiratory-tract infections (ARTI) [1]. It was found to be associated with ARTI in children, at a detection rate of 2–19% [2]–[5]. Three additional human bocaviruses, HBoV2, 3 and 4, discovered in human stool samples, have since been phylogenetically and serologically characterized [6]–[9]. However, whether these are associated with any diseases is currently unknown. HBoV1 is commonly detected in association with other respiratory viruses, and is the fourth most common respiratory virus (after respiratory syncytial virus (RSV), adenovirus and rhinovirus) in infants less than 2 years of age who are hospitalized for the treatment of acute wheezing [2], [10]–[12]. Indeed, ARTI is one of the leading causes of hospitalization of young children in developed countries [13], [14]. Acute HBoV1 infection, diagnosed by a virus load of >10^4^ genome copies (gc)/ml in respiratory samples, viraemia, or by detection of HBoV1-specific IgM or of an increase in the levels of IgG antibodies, results in respiratory illness [2], [15]–[20]. Recent descriptions of life-threatening HBoV1 infections in pediatric patients in association with high virus loads or diagnostic HBoV1-specific antibodies [21]–[23], in addition to a recent longitudinal study of children from infants to puberty, documenting a clear association of acute primary HBoV1 infection with respiratory symptoms [24], strongly support that HBoV1 is an etiological agent of both upper and lower ARTI.

HBoV1 has been classified as a new member of the genus *Bocavirus* of the family *Parvoviridae* [25], of which bovine parvovirus (BPV1) and minute virus of canines (MVC) are the prototypes [26], [27]. In comparison with the BPV1 and MVC genomes, the HBoV1 genome sequences obtained previously appeared to exclude the two termini, and therefore, were incomplete [28]. However, sequencing of the head-to-tail junctions of HBoV1 and HBoV3 “episomes,” which had been amplified in DNA samples extracted from HBoV1-infected differentiated human epithelial cells and from intestinal biopsies of HBoV3-infected patients, respectively, revealed portions of the HBoV termini [29], [30]. Notably, these sequences were conserved with the terminal sequences of BPV1 and MVC [28].

*In vitro* HBoV1 infection has been reported only once in well-differentiated human airway epithelia (HAE) [31]. That study provided only minimal information on virus replication, and did not include observations of pathophysiology. Obviously, the lack of a sustainable and highly reproducible system that enables high-yield virus production, as well as the ability to conduct reverse genetics is a significant barrier to further elucidation of HBoV1 replication and pathogenesis. In the current study, we have successfully sequenced the full-length HBoV1 genome and cloned it in a plasmid referred to as pIHBoV1. Furthermore, we have demonstrated that transfection of human embryonic kidney 293 (HEK293) cells with pIHBoV1 results in efficient production of HBoV1 virions at a high titer, and that these virions are able to productively infect both primary and conditionally transformed polarized HAE.

## Results

### The terminal hairpins of the HBoV1 genome are typical of those of the genus *Bocavirus*

**Figure 1:**
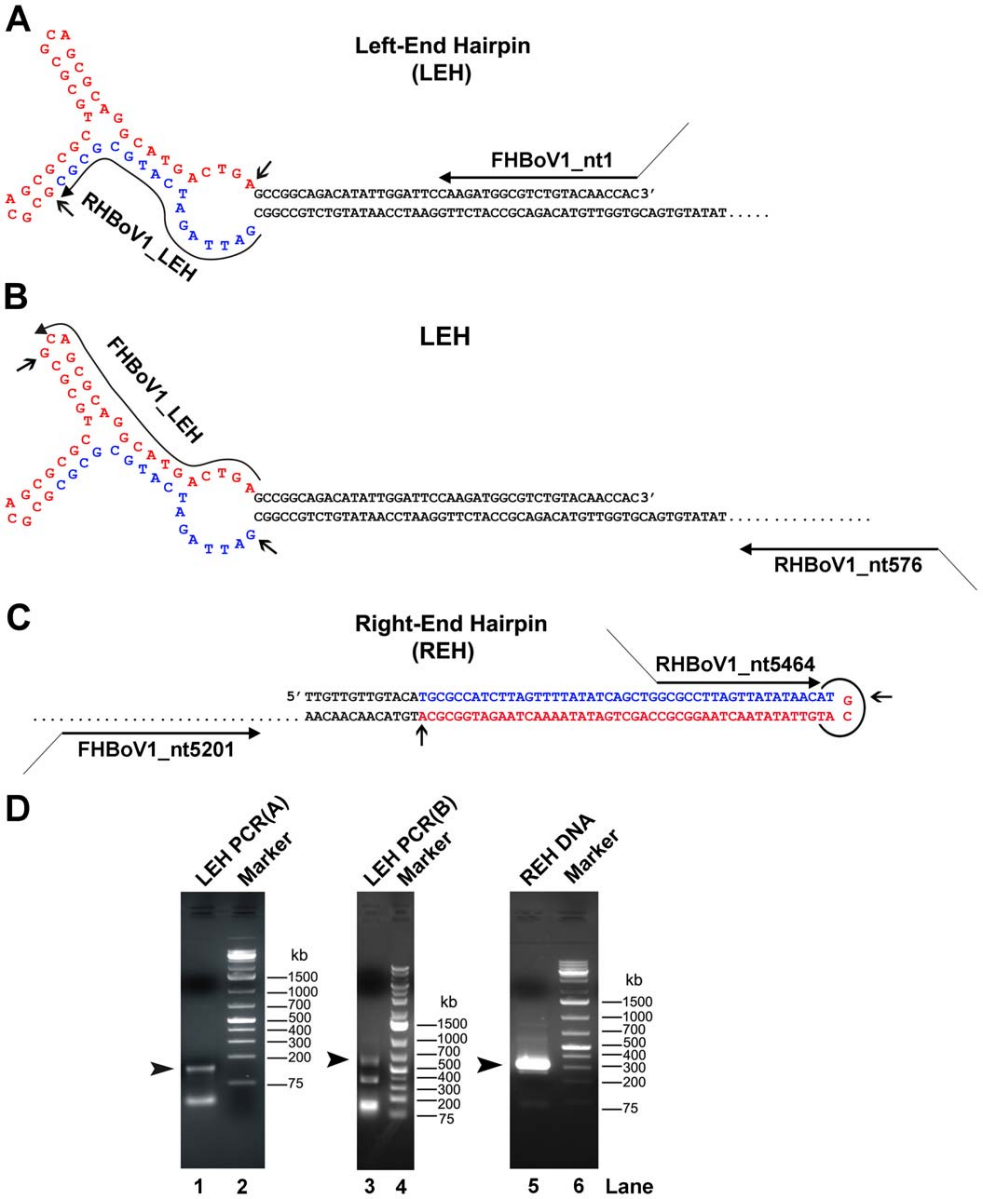
Sequencing the terminal hairpins of the HBoV1 Salvador1 isolate. Sequence and predicted structure of the left-end, LEH (**A&B**), and right-end, REH (**C**), hairpins are shown and diagramed, with PCR primers indicated by arrowed lines. PCR products were analyzed by electrophoresis on 2% agarose gels; the expected DNA bands are indicated by arrowheads (**D**). In both the LEH and REH, nucleotides in red represent new sequences identified in this study, nucleotides in blue represent sequences identified from the head-tail junction of an HBoV1 episome [28], [29], and nucleotides in black are the 5′end and 3′end sequences of the incomplete HBoV1 genome (GenBank: JQ411251).

A head-to-tail junction of an HBoV1 episome identified in an HBoV1-infected HAE [28], [29] was found to possess two sequences (3′-CGCGCGTA-5′ and 3′-GATTAG-5′) identical to parts of the BPV1 left-end hairpin (LEH) [27], [32]. This finding suggested that the head sequence is part of the HBoV1 LEH (nucleotides in blue; Figure 1A). We therefore used the head sequence as the 3′ end of a reverse primer (RHBoV1_LEH). Together with a forward primer (FHBoV1_nt1), which anchors the 3′ end of the HBoV1 genome predicted from the BPV1 LEH, we amplified the hairpin of the LEH from a viral DNA extract (1.2×10^8^ gc/ml) prepared from a nasopharyngeal aspirate taken from an HBoV1-infected patient (HBoV1 Salvador1 isolate) [17]. Only one specific DNA band was detected at approximately (∼)150-bp (Figure 1D, lane 1). Sequencing of this DNA revealed a novel sequence of the HBoV1 LEH (nucleotides in red between the two arrows; Figures 1A and S1A). Because the LEHs of the prototype bocaviruses BPV1 and MVC are asymmetric [27], [32], we set up another PCR reaction with a forward primer located in the hairpin (FHBoV1_LEH) and a reverse primer targeting a sequence downstream of the LEH at nt 576 (RHBoV1_nt576; Figure 1B). Sequencing of a DNA fragment (Figure S1B), detected as expected as a ∼600-bp band (Figure 1D, lane 3), confirmed the presence of the novel joint sequence and the LEH (Figure 1B).

The tail of the HBoV1 head-to-tail junction [28], [29] was found to contain a sequence (5′-GCG CCT TAG TTA TAT ATA ACA T-3′) identical to that of the right-end hairpin (REH) of the other prototypic bocavirus MVC [27]. We thus speculated that the entire HBoV1 REH is similar in structure to its MVC counterpart. Using a reverse primer targeted to this sequence (RHBoV1_nt5464) and a forward primer located upstream of the REH (FHBoV1_nt5201), we were able to amplify a specific ∼300-bp-long DNA fragment (Figure 1D, lane 5). Sequencing confirmed the presence of the palindromic hairpin of the predicted REH (nucleotides in red; Figures 1C and S1C), and revealed two novel nucleotides at the end of the hairpin (GC in red; Figure 1C).

These results indicate that we have identified, for the first time, both the LEH and REH of the HBoV1 genome from a clinical specimen, and confirm that the HBoV1 genome structure is typical of the genus *Bocavirus*.

### A full-length HBoV1 clone (pIHBoV1) is capable of replicating and producing progeny virus in HEK293 cells

We also cloned and sequenced the non-structural (NS) and capsid (VP) protein-coding (*NSVP*) genes of the HBoV1 Salvador1 isolate from the patient-extracted viral DNA. We then ligated the LEH, *NSVP* genes and REH into pBBSmaI using strategies diagramed in Figure S2, and refer to this full-length clone as pIHBoV1. We have deposited the sequence of the full-length genome of the isolate in GenBank (JQ923422).

**Figure 2:**
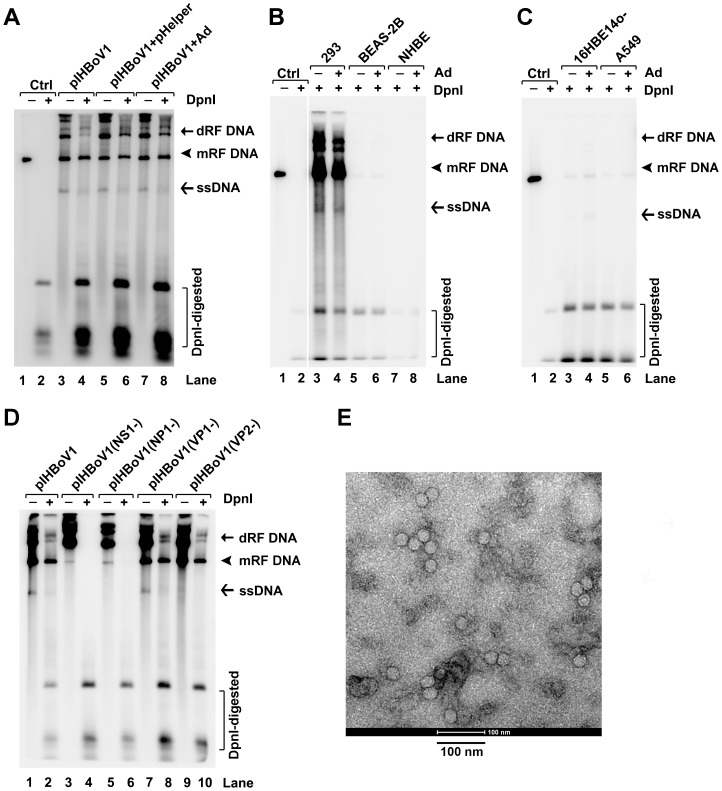
Southern blot analysis of pIHBoV1 transfection and electron microscopy analysis of purified virus. (**A–D**) Southern blot analysis. (**A**) HEK293 cells (plus or minus infection with adenovirus type 5 (Ad) at an MOI of 5) were transfected with pIHBoV1, alone or together with pHelper, as indicated. (**B&C**) pIHBoV1 was transfected into various cell lines that had (+) or had not (−) been infected with Ad as indicated. Lanes 1–8 in panel B were analyzed on the same gel, and the gels shown in panels B&C were transferred and blotted together. Ten ng of the HBoV1 dsDNA genome (∼5.6-kb), excised from pIHBoV1 using the SalI and XhoI sites, was used as a control (Ctrl) for DpnI digestion in panels A–C. (**D**) HEK293 cells were transfected with pIHBoV1 and its various mutants as indicated. At 48 h post-transfection, Hirt DNA was extracted and digested with (+) or without (−) DpnI, followed by Southern blotting using the HBoV1 dsDNA genome as a probe. dRF DNA, double replicative form DNA; mRF DNA, monomer replicative form DNA. (**E**) Negative staining electron micrograph. Purified HBoV1 particles were negatively stained and examined by a transmission electron microscopy. Bar indicates 100-nm.

As we previously showed that HEK293 cells support replication of the DNA of an autonomous human parvovirus (B19V) in the presence of adenovirus helper genes or adenovirus [33], we first investigated whether the adenovirus helper function is necessary for pIHBoV1 replication in HEK293 cells. Specifically, we transfected pIHBoV1 into HEK293 cells (untreated or infected with adenovirus), alone or with pHelper. Interestingly, we found that pIHBoV1 replicated well in the absence of helper virus. Indeed, all the three representative forms of replicated bocavirus DNA [27], [34] (DpnI digestion-resistant dRF DNA, mRF DNA and ssDNA) were detected in each test case, and at similar levels (Figure 2A). DpnI digestion-resistant DNA bands are newly replicated DNA in cells as DpnI digestion only cleaves plasmid DNA prepared from prokaryotic cells, which is methylated at the dam site [35]. In contrast, these DNA forms of the viral genome were absent in pIHBoV1-transfected primary airway epithelial cells (NHBE; Figure 2B, lanes 7&8) and present at very low levels (over 20 times lower than in pIHBoV1-transfected HEK293 cells) in pIHBoV1-transfected human airway epithelial cell lines BEAS-2B (Figure 2B, lanes 5&6), A549 and 16HBE14o- (Figure 2C), even in the presence of adenovirus. Thus, replication in these cells appears to be non-existent or poor in these contexts.

To confirm the specificity of DNA replication and the identity of the DpnI-resistant DNA bands, we disrupted the ORFs encoding viral proteins NS1, NP1, VP1 and VP2 in pIHBoV1; knockout of expression of the corresponding viral protein was confirmed by Western blot analysis. When the NS1 ORF was disrupted, no DpnI digestion-resistant DNA was detected (Figure 2D, lane 4), confirming that replication of this DNA requires NS1. Notably, when the NP1 ORF was disrupted, an RF DNA band was detected but it was very weak (Figure 2D, lane 6), suggesting that NP1 is also involved. When the VP2 ORF was knocked out, the ssDNA band disappeared, but this was not the case when VP1 was disrupted (VP2 was still expressed; Figure 2D, compare lanes 7 to 9), these findings are consistent with a role for the capsid formation in packaging of the parvoviral ssDNA genome [36]–[38].

The presence of the ssDNA band in pIHBoV1-transfected HEK293 cells suggested that progeny virions were produced. To prove this, we carried out large-scale pIHBoV1 transfection and CsCl equilibrium centrifugation to purify the virus that was produced. We fractionated the CsCl gradient, and found the highest HBoV1 gc (1–5×10^8^ gc/µl) at a density of 1.40 mg/ml, which is typical of the parvovirus virion. Electron microscopy analysis revealed that purified virus displayed a typical icosahedral structure, with a diameter of ∼26 nm (Figure 2E).

Collectively, these findings confirm that we have generated a full-length clone of HBoV1 capable of replicating and producing progeny virus in transfected HEK293 cells.

### HBoV1 progeny virus produced from pIHBoV1-transfected cells is infectious

**Figure 3:**
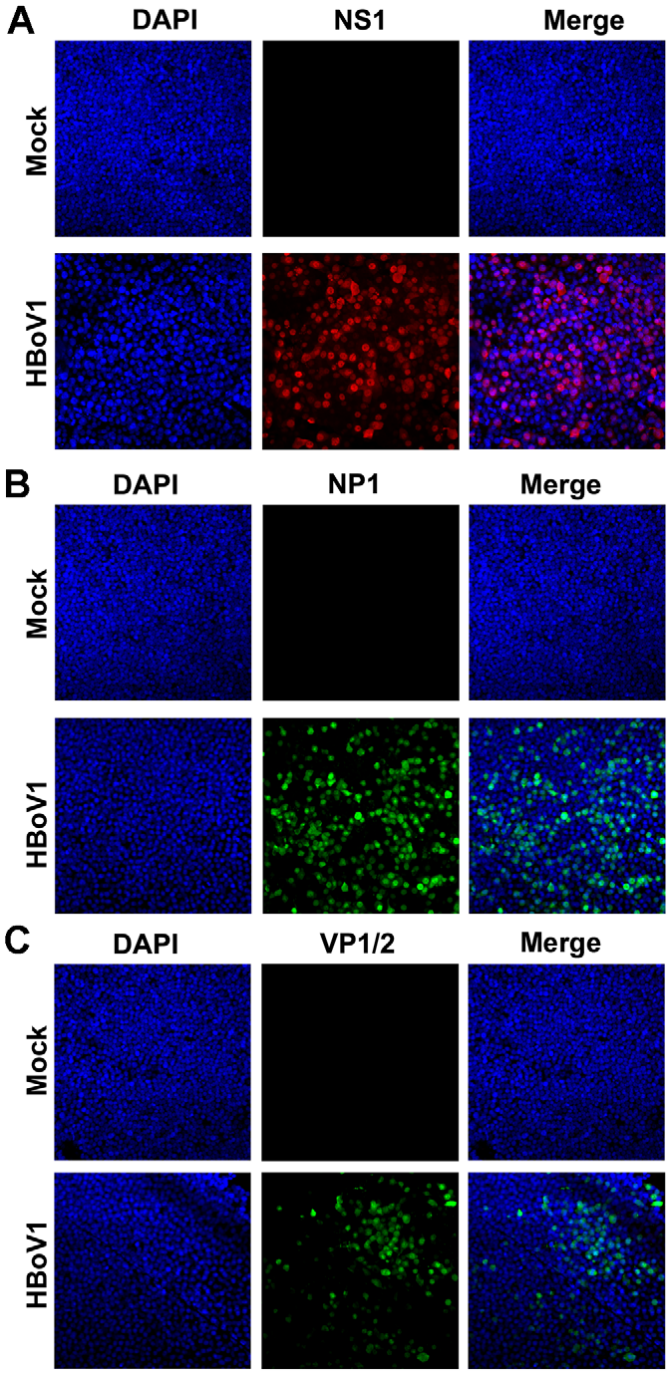
Transfection-produced HBoV1 infects primary B-HAE. The primary B-HAE (B31-11) was cultured in Millicell inserts, and infected with purified HBoV1 at the apical surface. At 12 days post-infection (p.i.), mock- and HBoV1-infected primary B-HAE cultures were fixed and analyzed by immunofluorescence (IF) using anti-(HBoV1) NS1 (**A**), NP1 (**B**), and VP1/2 (**C**) as indicated. Nuclei were stained with 4′,6-diamidino-2-phenylindole (DAPI; blue) and cells were visualized by confocal microscopy at a magnification of ×40. HAE, human airway epithelia.

The infectivity of the HBoV1 virions purified from pIHBoV1-transfected HEK293 cells was examined in polarized primary HAE, the *in vitro* culture model known to be permissive to HBoV1 infection [31]. Three sets (different donors, culture lots #B29-11, B31-11 and B33-11) of B-HAE were generated, and these were infected with HBoV1 from the apical side. Initially the B-HAE cultures were infected with various amounts of virus, and when a multiplicity of infection (MOI) of ∼750 gc/cell was used, most of the cells (∼80%) were positive for anti-NS1 staining (indicating that the viral genome had replicated and that genes encoded by it had been expressed) at 5 days post-infection (p.i.). This MOI was subsequently used for apical infection. Notably, B29-11, B31-11 and B33-11 HAE each supported productive HBoV1 infection (Figures 3 and S3). Immunofluorescence (IF) analysis of infected B31-11 HAE at 12 days p.i. showed that virtually all the cells expressed NS1 and NP1 (Figures 3A and 3B), and that a good portion of the infected cells expressed capsid proteins (VP1/2; Figure 3C).

**Figure 4:**
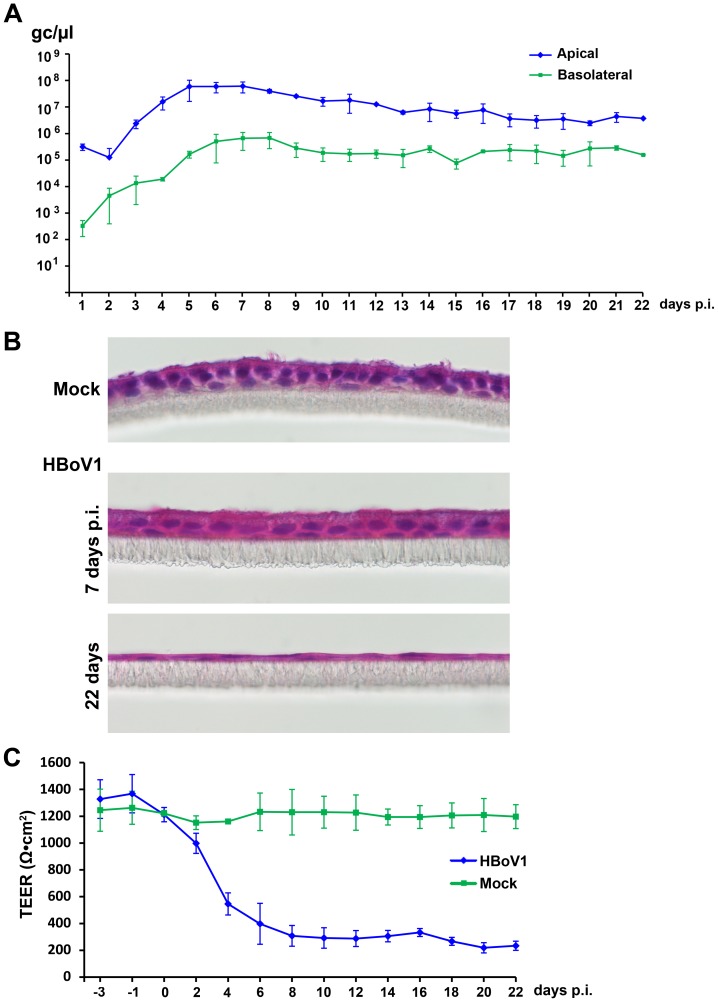
HBoV1 infection of primary B-HAE is persistent and causes cytopathogenic effects. The primary B-HAE (B33-11) was cultured in Millicell inserts, and infected with HBoV1 at the apical surface. (**A**) At the indicated days p.i., the apical surface was washed with 200 µl of PBS to remove released virus. 50 µl of the medium were taken from the basolateral side. DNase-resistant HBoV1 DNA copies were quantified by qPCR (y-axis) and plotted to the days p.i. as shown. Averages and standard deviations are shown. (**B**) At 7 and 22 days p.i., HBoV1-infected primary B-HAE membranes taken from the bottom of the inserts were embedded in OCT, sectioned, and stained using hematoxylin and eosin. Mock-infected B-HAE was taken at 22 days p.i. Images were taken at a magnification of ×60. (**C**) The transepithelial electrical resistance (TEER) of mock- and HBoV1-infected primary B-HAEs was measured using an epithelial Volt-Ohm Meter (Millipore) at the indicated days p.i. Averages and standard deviations are shown.

The production of progeny virus following HBoV1 infection was monitored daily by collecting samples from both the apical and basolateral chambers of the HAE culture and carrying out HBoV1-specific quantitative PCR (qPCR; Figures 4A and S3B). In the case of B33-11 B-HAE, apical release was obviously initiated at 3 days p.i., then continued to increase to a peak of ∼10^8^ gc/µl at 5–7 days p.i., then decreased slightly through day 10 p.i. and was maintained at a level of ∼10^7^ gc/µl through day 22 p.i. (Figure 4A). The total virus yield from one Millicell insert of 0.6 cm^2^ over a 24-h interval was greater than 2×10^10^ gc. This result suggested that productive HBoV1 infection of primary B-HAE is persistent. Notably, in the B-HAE cultures from both donors, virus was also continuously released from the basolateral side, keeping pace with apical secretion throughout, though at levels about one log lower than the release from the apical surface (Figures 4A and S3B). The genomes of the progeny virions released from infected B-HAE were amplified and sequenced using the primers listed in Figure 1 and primers spanning the *NSVP* genes between the termini. The result showed an identical sequence with that of the HBoV1 Salvador isolate (Genbank JQ923422). Additionally, no virus was detected in mock-infected B-HAE (data not shown).

Taken together, these results demonstrate that the HBoV1 virions produced by pIHBoV1 transfection is capable of infecting polarized primary HAE cultures from cells derived from various donors and releasing identical progeny virions from infected primary HAE. More importantly, we found that productive HBoV1 infection was persistent.

### HBoV1 infection of primary B-HAE features characteristics of respiratory-tract injury

Although no gross cytopathic effects were observed in HBoV1-infected B-HAE, histology analysis of mock- vs. HBoV1-infected epithelia (B33-11) revealed morphological differences: infected B-HAE did not feature obvious cilia at 7 days p.i., and was significantly thinner than the mock-infected one on average at 22 days p.i. (Figure 4B). We further monitored the transepithelial electrical resistance (TEER) during infection of B-HAE, and found that at 6 days p.i., it was reduced from a value of ∼1,200 to <400 Ω.cm^2^, while the mock-infected B-HAE maintained the initial TEER (Figure 4C). Notably, the decrease in TEER in the infected B-HAE was accompanied by an increase in HBoV1 secretion (Figure 4A).

**Figure 5:**
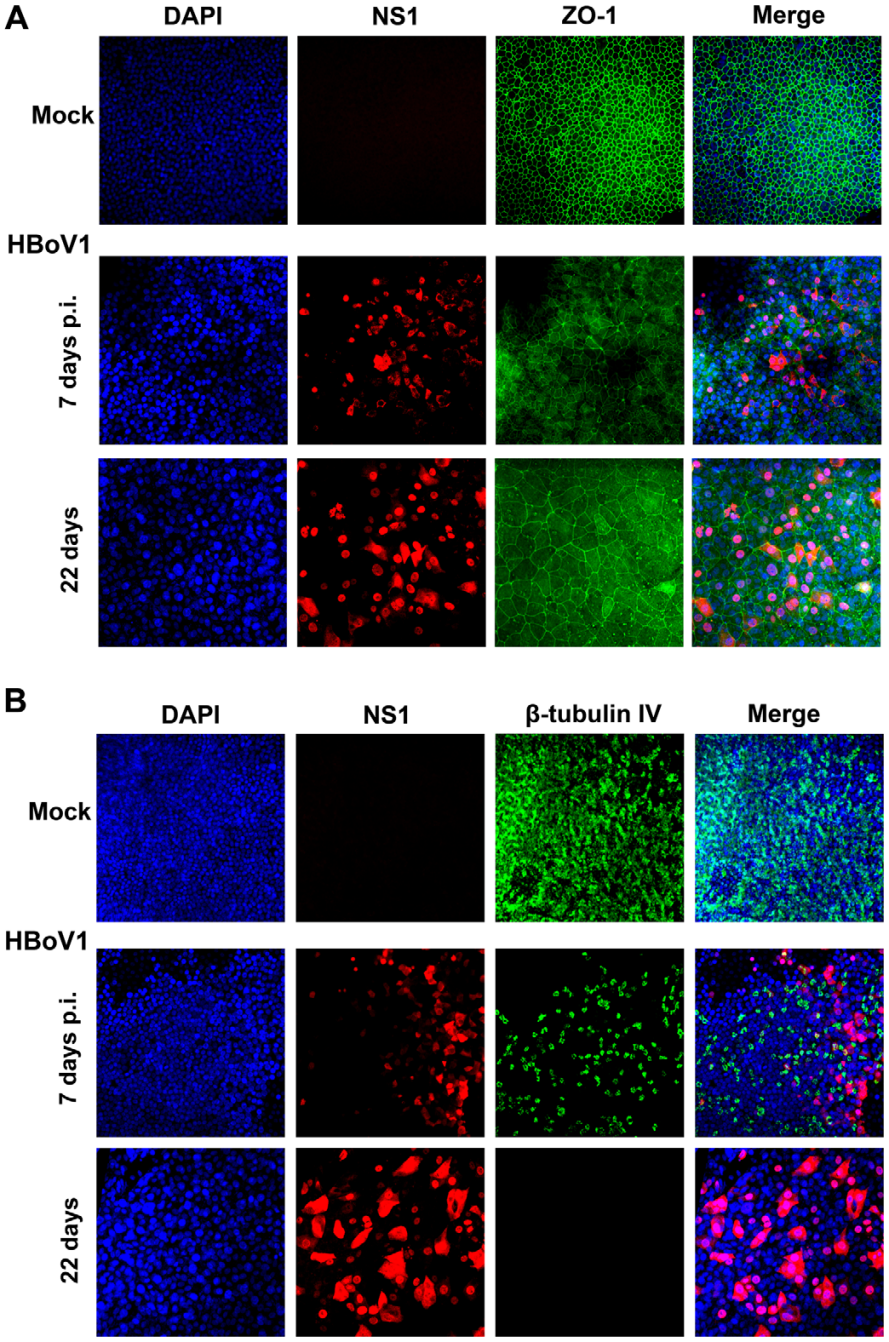
IF analysis of the tight junction protein ZO-1 and the cilia marker β-tubulin IV during HBoV1 infection of primary B-HAE. Mock- and HBoV1-infected B-HAE (B33-11) cultures at the indicated days p.i. were co-stained with anti-NS1 and anti-ZO-1 (Invitrogen) antibodies (**A**), or co-stained with anti-(HBoV1) NS1 and anti-β-tubulin IV (Sigma) antibodies (**B**). Confocal images were taken at a magnification of ×40. Nuclei were stained with DAPI (blue).

To confirm a role for HBoV1 infection in disruption of the barrier function of the epithelium, we examined the distribution of the tight junction protein Zona occludens-1 (ZO-1) [39]. Infected B-HAE showed dissociation of ZO-1 from the periphery of cells started from 7 days p.i., compared with mock-infected B-HAE (Figure 5A), which likely plays a role in reducing TEER. Cumulatively, these results demonstrate that HBoV1 infection disrupts the integrity of HAE and that this may involve breakdown of polarity and redistribution of the tight junction protein ZO-1. To confirm a role for HBoV1 infection in the loss of cilia, we examined expression of the β-tubulin IV, which is a marker of cilia [40], [41]. In HBoV1-infected B-HAE, expression of β-tubulin IV was drastically decreased at 7 days p.i., and was not detected at 22 days p.i., in contrast to that in mock-infected B-HAE (Figure 5B). These results confirmed that HBoV1 infection caused the loss of cilia in infected B-HAE. Notably, infected B-HAE showed changes of nuclear enlargement, which became obvious at 22 days p.i. (Figure 5, DAPI), indicating airway epithelial cell hypertrophy.

Collectively, we found that productive HBoV1 infection disrupted the tight junction barrier, lead to the loss of cilia and airway epithelial cell hypertrophy. These are hallmarks of respiratory tract injury when a loss of epithelial cell polarity occurs.

### An immortalized human airway epithelial cell line supports HBoV1 infection when the cells are polarized

**Figure 6:**
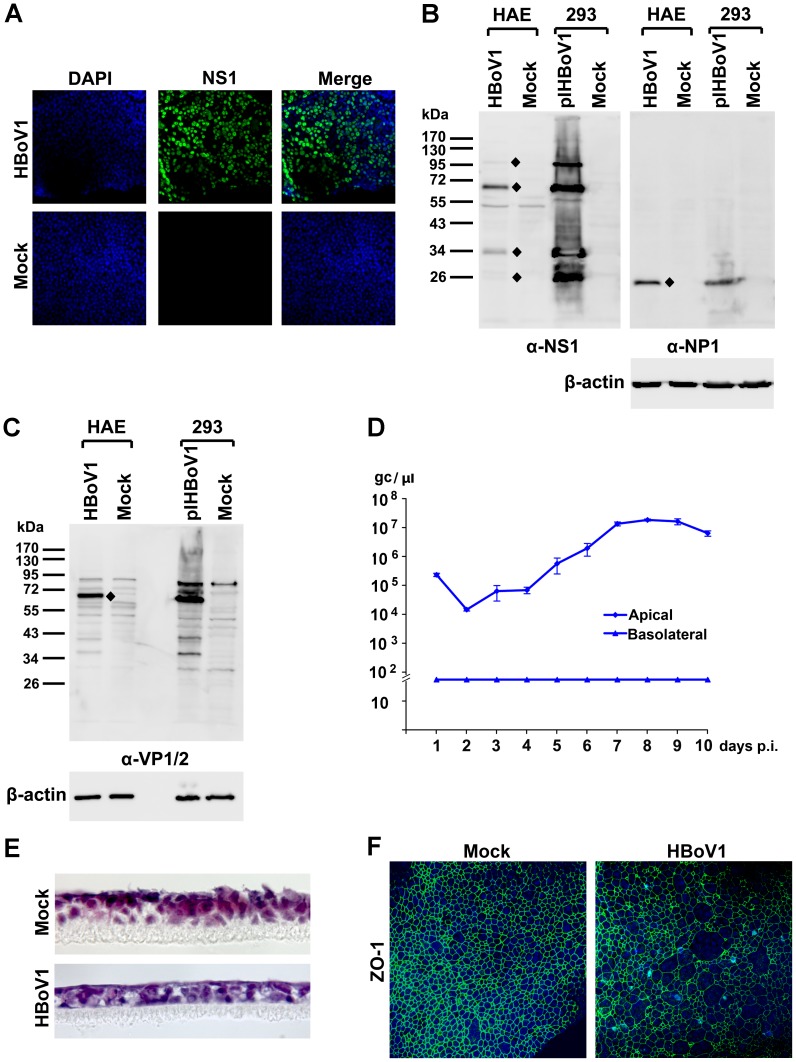
Analyses of HBoV1-infected CuFi-HAE. (**A**) At 10 days p.i., mock- and HBoV1-infected CuFi-HAEs were analyzed by IF with an anti-(HBoV1)NS1 antibody. (**B&C**) At 5 days p.i., mock- and HBoV1-infected CuFi-HAEs were analyzed by Western blotting with anti-(HBoV1)NS1, NP1, and VP1/2 antibodies as indicated. pIHBoV1-transfected HEK293 cells were used as controls. (**B**) The blot was reprobed sequentially with anti-NP1 and anti-β-actin antibodies; (**C**) the same samples were separated and blotted with the anti-β-actin antibody. Diamonds indicate specific viral proteins detected, and the sizes of the marker are shown. (**D**) CuFi-HAE was infected with HBoV1, virus was collected from both the apical and basolateral sides at days p.i. as indicated, and quantified. Averages and standard deviations are shown. (**E**) At 10 days p.i., mock- and HBoV1-infected CuFi-HAE membranes were stained with hematoxylin and eosin. Images were taken at a magnification of ×60. (**F**) At 10 days p.i., mock- and HBoV1-infected CuFi-HAEs were analyzed by IF with an anti-ZO-1 antibody (BD Bioscience). Nuclei were stained with DAPI. Confocal images in panels A and F were taken at a magnification of ×40.

Although primary HAE cultures support HBoV1 infection, their usefulness is limited by the variability between donors, tissue availability and high cost. We thus explored alternative cell culture models for their abilities to support HBoV1 infection. Using the purified HBoV1, we examined HEK293 cells, other common epithelial cell lines permissive to common respiratory viruses [42], including HeLa, MDCK, MRC-5, LLC-MK2 and Vero-E6, and several transformed or immortalized human airway epithelial cell lines (A549, BEAS-2B, 16HBE14o- [43], NuLi-1 and CuFi-8 [44]), as well as primary NHBE cells for the ability to support infection in conventional monolayer culture. All were negative for HBoV1 infection as determined by IF analysis (data not shown). We next speculated that since some respiratory viruses infect polarized HAE but not undifferentiated cells [45], some characteristics of the polarized epithelia may be critical for HBoV1 infection. We thus polarized immortalized cells (NuLi-1 and CuFi-8) at an air-liquid interface (ALI) for one month. Once polarization was confirmed by detection of a TEER of >500 Ω.cm^2^, the cultures were infected with HBoV1, under the same conditions as used for primary B-HAE cultures. Notably, IF analysis revealed that at 10 days p.i., HBoV1-infected CuFi-HAE (differentiated from CuFi-8 cells) was uniformly positive for NS1 (Figure 6A), whereas the HBoV1-infected NuLi-HAE (differentiated from NuLi-1 cells) was not (Figure S4). Moreover, the CuFi-HAE did express HBoV1 NS1, NP1 and VP1/VP2 proteins (Figures 6B and 6C). The kinetics of virus release from the apical surface was similar to that of a primary B-HAE infected with virus at a similar titer (maximally 2×10^7^ gc/µl), although virus release from the basolateral surface was undetectable (Figure 6D). HBoV1 infection also resulted in a decrease in the thickness of the epithelium (Figure 6E), and dissociation of the tight junction protein ZO-1 from the epithelial cell peripheries (Figure 6F).

Collectively, these findings demonstrate that the immortalized cell line CuFi-8 [44], when cultured and polarized at an ALI, supports HBoV1 infection, and recapitulates the infection phenotypes observed in primary HAE, including destruction of the airway epithelial structure.

## Discussion

In this study, we have cloned the full-length HBoV1 genome and identified its terminal hairpins. Virions produced from transfection of this clone into HEK293 cells are capable of infecting polarized HAE cultures. Thus, we have established a reverse genetics system that overcomes the critical barriers to studying the molecular biology and pathogenesis of HBoV1, using an *in vitro* culture model system of HAE.

It is notable that the HBoV1 terminal hairpins appear to be hybrid relicts of the prototype bocavirus BPV1 at the LEH, but of MVC at the REH [28]. Replication of HBoV1 DNA in HEK293 cells revealed typical replicative intermediates of parvoviral DNA. Although the head-tail junctions are unexpected in the replication of autonomous parvoviruses, they were likely generated during the cycle of rolling hairpin-dependent DNA replication [46]. Therefore, we believe that the replication of HBoV1 DNA basically follows the model of rolling hairpin-dependent DNA replication of autonomous parvoviruses, with terminal and junction resolutions at the REH and LEH, respectively [46]. The replication of parvoviral DNA depends on entry into S phase of the cell cycle or the presence of helper viruses [46], [47]. In this regard, it is puzzling that mature, uninjured airway epithelia are mitotically quiescent (<1% of cells dividing) [48]–[50], as are the majority of the cells in polarized HAE (in the G0 phase of the cell cycle). However, recombinant adeno-associated virus (AAV; in genus *Dependovirus* of the family of *Parvoviridae*) infects HAE apically and expresses reporter genes [51]–[53]. Gene expression by recombinant AAV requires a conversion of the ssDNA viral genome to a double-stranded DNA form that is capable to be transcribed [54]. This conversion involves DNA synthesis. Hence, we hypothesize that HBoV1 employs a similar approach to synthesize its replicative form DNA. Notably, wild type AAV infected primary HAE apically and replicated when adenovirus was co-infected [55]. The exact mechanism of how HBoV1 replicates in normal HAE will be an interesting topic for further investigation.

The airway epithelium, a ciliated pseudo-stratified columnar epithelium, represents the first barrier against inhaled microbes and actively prevents the entry of respiratory pathogens. It consists of ciliated cells, basal cells and secretory goblet cells that together with the mucosal immune system, provide local defense mechanisms for the mucociliary clearance of inhaled microorganisms [56]. The polarized ciliated primary HAE, which is generated by growing isolated tracheobronchial epithelial cells at an ALI for on average one month, forms a pseudo-stratified, mucociliary epithelium and displays morphologic and phenotypic characteristics resembling those of the *in vivo* human cartilaginous airway epithelium of the lung [57], [58]. Recent studies have revealed that this model system recapitulates important characteristics of interactions between respiratory viruses and their host cells [41], [45], [59]–[62].

In the current study, we have examined primary B-HAE cultures obtained from three different donors. HBoV1 infection of primary B-HAE was persistent and caused morphological changes of the epithelia, i.e. disruption of the tight barrier junctions, loss of cilia and epithelial cell hypertrophy. The loss of the former, plasma membrane structures that seal the perimeters of the polarized epithelial cells of the monolayer, is known to damage the cell barrier necessary to maintain vectorial secretion, absorption and transport. ZO-1, which we monitored here, is specifically associated with the tight junctions and remains the standard marker for these structures. Similarly, cilia play important roles in airway epithelia, in that they drive inhaled particles that adhere to mucus secreted by goblet cells outward [63]. HBoV1 infection compromises barrier function, and thus potentially increases permeability of the airway epithelia to allergens and susceptibility to secondary infections by microbes. The observed shedding of virus from the basolateral surface of infected primary HAE, albeit at a lower level (∼1 log lower than that from the apical surface), is consistent with the facts that HBoV1 infection disrupted the polarity of the pseudo-stratified epithelial barrier and resulted in the leakage to the basolateral chamber. This explanation is also supported by HBoV1 infection of CuFi-HAE, where disruption of the tight junction structure was less severe and virus was released only from the apical membrane. The induction of leakage by HBoV1 also suggests a mechanism that accounts for the viraemia observed in HBoV1-infected patients [5]. Further disease pathology could be accounted for by infection-induced loss of cilia of the airway epithelia; a lack of cilia is often responsible for bronchiolitis [64]–[66]. Therefore, our study provides direct evidence that HBoV1 is pathogenic to polarized HAE, which serves as *in vitro* model of the lung [57], [58]. Since HBoV1 is frequently detected with other respiratory viruses in infants hospitalized for acute wheezing [2], [10]–[12], the apparent pathological changes observed in HBoV1-infected HAE suggest that prior-infection of HBoV1 likely facilitates the progression of co-infection-driven pathogenesis in the patient.

The kinetics of virus release from the apical chamber of HAE infected with the progeny virus of pIHBoV1 (cloned from the clinical Salvador1 isolate) was similar to that following infection with the HBoV1 Bonn1 isolate, a clinical specimen [31]. We believe that our study of HBoV1 infection of primary HAE reproduces infection of the virus from clinical specimens. In addition, we generated virus from a pIHBoV1-b clone, which contains the *NSVP* genes from the prototype HBoV1 st2 isolate [1]. Infection of primary B-HAE with this st2 virus resulted in a level of virus production similar to that observed here using the Salvador1 isolate (data not shown). We believe that our study with the laboratory-produced HBoV1 Salvador1 represents infection of HBoV1 of clinical specimens in HAE. The MOI used for infection in the current study was high. However, it should be noted that this titer is based on the physical numbers of virion particles as there are no practical methods for determining the infectious titer of HBoV1 preparations. It should also be taken into consideration that extensive parvovirus inactivation occurs during the purification process, i.e. during CsCl equilibrium ultracentrifugation [67]. Virus infection of HAE most likely reflects HBoV1 infection of the lung airways in patients with a high virus load in respiratory secretions [5].

The fact that pIHBoV1 did not replicate well in undifferentiated human airway epithelial cells (Figures 2B and 2C) indicates that polarization and differentiation of the HAE is critical for HBoV1 DNA replication. Nevertheless, polarized NuLi-HAE, which is derived from normal human airway epithelial cells, did not support HBoV1 infection, but the CuFi-HAE derived from airway epithelial cells isolated from a cystic fibrosis patient did. The CuFi-HAE is unique relative to the others in that it retains the capacity to develop epithelia that actively transport in Na^+^ but not Cl^−^ because of the mutation in the cystic fibrosis gene [44]. Given the high complexity of the airway epithelium, we speculate that the permissiveness of HBoV1 infection is dependent on various steps of virus infection, e.g. attachment, entry, intracellular trafficking, and DNA replication of the virus. Nevertheless, a polarized CuFi-HAE model derived from the CuFi-8 cell line represents a novel stable cell culture model that is providing unexpected insights into the infection characteristics of HBoV1. Although HBoV1 infection of CuFi-HAE reproduced disruption of the barrier tight junctions like that seen also in primary B-HAE, the absence of virus on the basolateral side implies that in HAE the secretion of HBoV1 is apically polarized. We speculate that the milder damage of tight junctions in these cells might prevent virus release from the basolateral side of infected CuFi-HAE. Further studies will focus on understanding the permissiveness of CuFi-HAE to HBoV1 infection and on the reason for the ease of infection of an HAE with a cystic fibrosis phenotype.

It has been shown that HBoV1 remains detectable in the upper airways of patients for weeks and months, even up to half a year [68]–[71]. However, the mechanism behind this persistence, i.e. whether it is due to persistent replication and shedding, passive persistence after primary infection, or recurrent mucosal surface contamination, has remained unknown. Our results in *in vitro* HAE cultures showed that HBoV1 is able to replicate and shed from both the apical and basolateral surfaces at least for three weeks, supporting the notion that shedding of the virus from the airways is a long-lasting process. This may further explain why a high rate of co-infection, or co-detection, between HBoV1 and other respiratory viruses has been reported [5]. Since recombinant AAV persists as an episome in transduced tissues, which prolongs gene expression [72], [73], it is possible that also the HBoV1 genome can be presented as an episome [29], [30] for long term expression and replication. Apparently, the mechanism underlying this feature of HBoV1 infection warrants further investigation. However, in contrast to the other human-pathogenic B19V, HBoV1 does not seem to persist in human tissues for many years [74].

In conclusion, our findings indicate that the innovative reverse genetics system for studying HBoV1 infection that we describe here will enable us to elucidate the mechanism of HBoV1 replication and pathogenesis in a polarized HAE. Our system mimics natural HBoV1 infection of the *in vivo* human cartilaginous airway epithelia. The pathogenesis of HBoV1 in co-infection with other respiratory viruses and in conditions of lung diseases is a focus of future study.

## Materials and Methods

### Cell culture

#### Cell lines and primary cells

Human embryonic kidney 293 (HEK293) cells (CRL-1573), HeLa (CCL-2) , MDCK (CCL-34), MRC-5 (CCL-171) , LLC-MK2 (CCL-7), and Vero-E6 (CRL-1586) were obtained from American Type Culture Collection (ATCC, Manassas, VA), and were cultured in Dulbecco's Modified Eagle Medium (DMEM) with 10% fetal calf serum (FCS). The cell lines originating from human airway epithelial cells are A549 (ATCC CCL-185), BEAS-2B (ATCC CRL-9609), 16HBE14o- (obtained from Dr. Dieter Gruenert; [43]), as well as NuLi-1 and CuFi-8 (Tissue and Cell Culture Core, Center for Gene Therapy, University of Iowa). NuLi-1 and CuFi-8 were immortalized from normal and cystic fibrosis human primary airway cells, respectively, by expressing hTERT and HPV E6/E7 genes [44]. Primary Clonetics normal human bronchial/tracheal epithelial cells (NHBE) were purchased from Lonza (Walkersville, MD). Cells were cultured in media following instructions provided by the supplier.

#### Human airway epithelium cultures

Polarized primary HAE, termed as primary B-HAE, was generated by growing isolated human airway (tracheobronchial) epithelial cells (three HAE cultures were generated from different donors) on collagen-coated, semipermeable membrane inserts (0.6 cm^2^, Millicell-PCF; Millipore, Billerica, MA), and then allowing them to differentiate at an air-liquid interface (ALI); this was carried out at the Tissue and Cell Culture Core of the Center for Gene Therapy, University of Iowa [44], [58], [75], [76]. After 3–4 weeks of culture at an ALI, the polarity of the HAE was determined based on the transepithelial electrical resistance (TEER) using an epithelial Volt-Ohm Meter (Millipore) and the relationship to infectability was assessed; a value of over 1,000 Ω.cm^2^ was required for HBoV1 infection. CuFi- and NuLi-HAE were generated following the same method as above, but using the immortalized airway epithelial cell lines, CuFi-8 and NuLi-1, respectively. The primary B-, CuFi-, and NuLi-HAE were cultured, differentiated and maintained in (50%∶50%) DMEM:F12 medium containing 2% Ultroser G (Pall BioSepra, Cergy-Staint-Christophe, France).

### Isolation of virus and extraction of viral DNA

A nasopharyngeal aspirate was obtained from a child with community-acquired pneumonia in Salvador, Brazil, who had an acute HBoV1 infection (seroconversion, viraemia, and over 10^4^ gc of HBoV1 per ml of aspirate) [17]. Viral DNA was extracted according to a method described previously [77].

### Primers used and sequence amplification by the Polymerase Chain Reaction (PCR)

The sequence of the head-to-tail junction of the HBoV1 episome suggests that HBoV LEH and REH share similarities both in structure and sequence with that of the BPV LEH and MVC REH, respectively [27], [29]. Based on this information [28], we designed primers to amplify the HBoV1 termini, which are shown in Table 1 and Figure 1. The Phusion high fidelity PCR kit (NEB, Ipswich, MA) was used following the manufactures' instructions, to amplify the left-end hairpin (LEH) and the right-end hairpin (REH) of HBoV1. Briefly, the DNA denaturation at 98°C for 30 s was followed by 35 cycles of: denaturing at 98°C for 10 s; annealing at 55°C for 15 s; and extension at 72°C for 30 s. Following the final cycle, extension was continued at 72°C for 10 min. The PCR products were analyzed by electrophoresis in a 2% agarose gel. DNA bands were extracted using the QIAquick gel extraction kit (Qiagen, Valencia, CA), and the extracted DNA was directly sequenced at MCLAB (South San Francisco, CA), using primers complementary to the extended sequences on the forward and reverse amplification primers. PCR-generated DNA was cloned in pGEM-T vector (Promega, Madison, WI), and DNAs isolated from cultures of individual clones were subsequently sequenced.

**Table 1:** Sequences of PCR primers designed for amplifying the terminal hairpins of HBoV1.

Name	Sequence (5′-3′)
FHBoV1_nt1	**GTATTTTCAGGGCCTCGTCGAC** GTGGTTGTACAGACGCCATCTTG
RHBoV1_LEH	**GGAAGGCCTTGGATGTGGAAAGGCCG** GATTAGATCATGCGCGC
FHBoV1_LEH	**CTAGGATCCGTATTTTCAGGGCCTCGTCGAC** TCAGTCATGCCTGCGCTG
RHBoV1_nt576	**CGCAAGCTTCTCGAGTCTAGA** AGCCCCAAAATGGCGATCTTCTAAAGA
FHBoV1_nt5201	**CTAGGATCC** GTTCCTCCTCAATGGACAAGCG
RHBoV1_nt5464	**CACTGCAAGCTTGGAAGGCCTTGGATGTGGAAGCCG** GCGCCTTAGTTATATAACAT

Note: Nucleotides underlined are HBoV1 sequences or sequences complementary to the HBoV1 sequence, and nucleotides shown in bold fonts are sequences containing restriction enzyme sites and random sequences used to optimize PCR reactions and cloning.

### Construction of a full-length HBoV1 clone and its mutants

#### Construction of the pBB vector

We first constructed a pBBSmaI vector by inserting a linker of 5′-SalI-SacII-KpnI-SmaI-ApaI-SphI-KpnI-HindIII-XhoI-3′ into a vector backbone (pProEX HTb vector; Invitrogen) generated from the B19V infectious clone pM20 [78] by removing all of the B19V sequence (SalI-digestion). All cloning work was carried out in the *Escherichia coli* strain of Sure 2 (Agilent, La Jolla, CA). All the nucleotide numbers of HBoV1 refer to the HBoV1 full-length genome (GenBank accession no.:JQ923422).

#### Cloning of the left-end hairpin (Figure S2B)

The DNA fragment SalI-BglII-nt93-518(BspEI)-576-XhoI-HindIII (containing the HBoV1 sequence nt 93–576), was amplified from the viral DNA and inserted into SaII/HindIII-digested pBBSmaI, to produce pBB2.1. Another DNA, SalI-nt1-86-BclI (containing HBoV1 nt 1–86 sequence), was synthesized according to the LEH sequence obtained in Figures 1A and 1B, and placed between the SalI and BglII sites in pBB2.1, with ligation of the BclI and BglII sites reproducing the HBoV1 sequence nt 87–92. The resultant plasmid harboring the 5′ HBoV1 nt 1–576 sequence with an intact LEH is designated pBB-LEH.

#### Cloning of the right-end hairpin (Figure S2C)

The DNA fragment SalI-nt4097-4139(BglII)-5427(KasI)-ApaI (containing the HBoV1 nt 4097–5427 sequence) was amplified from viral DNA and inserted into SaII/ApaI-digested pBBSmaI, resulting in pBB2.2. Another DNA fragment, ApaI-nt5460(KasI)-5543-XhoI (containing HBoV1 nt 5460–5543 sequence) was synthesized based on the REH sequence (Figure 1C) and placed between the ApaI and HindIII sequences in pBB2.2, resulting in pBB-REH(Δ5428–5459). The missing short fragment between the two KasI sites encompassing nt 5428–5459 was recovered by a synthesized KasI linker based on the REH sequence (Figure 1C) and inserted into KasI-digested pBB-REH(Δ5428–5459). The resultant plasmid harboring the 3′ HBoV1 nt 4097–5543 sequence with an intact REH is designated pBB-REH.

#### Cloning of the pIHBoV1 (Figure S2D)

The HBoV1 DNA fragment SalI-nt1-518(BspEI)-576-XhoI, which was obtained from SalI/XhoI-digestion of pBB-LEH, was ligated into SalI-digested pBB-REH, resulting in pBB-LEH(BspEI/BglII)REH. The larger fragment produced by digestion of this plasmid with BspEI/BglII was ligated to the HBoV1 DNA fragment nt 518(BspEI)-4139(BglII), which was amplified from the viral DNA. The final construct containing the full-length HBoV1 (nt 1–5543) was designated pIHBoV1.

#### Construction of pIHBoV1 mutants

pIHBoV1NS1(−) and pIHBoV1NP1(−) were constructed by mutating HBoV1 nt 542 from T to A, and nt 2588 from G to A, resulting in stop codons that lead to early termination of the NS1 and NP1 ORFs, respectively. Similarly, pIHBoV1VP1(−) and pIHBoV1VP2(−) were generated by mutating HBoV1 nt 3205 from T to A, and nt 3540 from T to G, disrupting VP1 and VP2 ORFs, respectively.

### Transfection

Cells grown in 60-mm dishes were transfected with 2 µg of plasmid as indicated in Figure 2; the Lipofectamine and Plus reagents (Invitrogen/Life Technologies, Carlsbad, CA) were used as previously described [79]. For some of the transfection experiments, HEK293 cells were cotransfected with 2 µg of pHelper plasmid (Agilent), which contains the adenovirus 5 (Ad5) E2a, E4orf6, and VA genes, or infected with adenovirus type 5 (Ad) at an MOI of 5 as previously described [79].

### Southern blot analysis

Low molecular weight (Hirt) DNA was extracted from transfected cells, digested with DpnI (or left undigested) and analyzed by Southern blotting as previously described [80].

### Western blot analysis

Cells were lysed, separated by SDS-8% polyacrylamide gel electrophoresis (PAGE), and blotted with antibodies as indicated as previously described [81].

### Production and purification of HBoV1

HEK293 cells were cultured on fifteen 150-mm plates in DMEM-10%FCS, and transfected with 15 µg of pIHBoV1 per dish using LipoD293 (SignaGen, Gaithersburg, MD). After being maintained for 48 h at 5% CO_2_ and 37°C, the cells were collected, resuspended in 10 ml of phosphate buffered saline, pH7.4 (PBS), and lysed by subjecting them to four freezing (−196°C) and thawing (37°C) cycles. The cell lysate was then spun at 10,000 rpm for 30 min. The supernatant was collected and assessed on a continuous CsCl gradient. In brief, the density was adjusted to 1.40 g/ml by adding CsCl, and the sample was loaded into an 11-ml centrifuge tube and spun in a Sorvall TH641 rotor at 36,000 rpm, for 36 h at 20°C.

Fractions of 550 µl (20 fractions) were collected with a Piston Gradient Fractionator (BioComp, Fredericton, NB, Canada), and the density of each was determined by an Abbe's Refractometer. Viral DNA was extracted from each fraction and quantified with respect to the number of HBoV1 gc, using HBoV1-specific qPCR as described below. Those fractions containing the highest numbers of HBoV1 gc were dialyzed against PBS, and then viewed by electron microscope and used to infect HAE cultures.

### Observation by electron microscopy (EM)

The final purified virus preparation was concentrated by ∼5-fold, and adsorbed for 1 min on a 300-mesh copper EM grid coated with a carbon film, followed by washing with deionized water for 5 s and staining with 1% uranyl acetate for 1 min. The grid was air dried, and was inspected on a 200 kV Tecnai F20 G2 transmission electron microscope equipped with a field emission gun.

### Virus infection

Fully differentiated primary B- (each of the three distinct subtypes), CuFi- and NuLi-HAE were cultured in Millicell inserts (0.6 cm^2^; Millipore) and inoculated with 150 µl of purified HBoV1 (1×10^7^ gc/µl in phosphate buffered saline, pH7.4; PBS) from the apical surface (at a multiplicity of infection, MOI, of ∼750 gc/cell; an average of 2×10^6^ cells per insert). For each of the HAE, a 2-h incubation was followed by aspiration of the virus from the apical chamber and by three washes of the cells with 200 µl of PBS to remove unbound virus. The HAEs were then further cultured at an ALI.

For conventional monolayer cells, cells cultured in chamber slides (Lab-Tek II; Nalge Nunc) were infected with purified HBoV1 at an MOI of 1,000 gc/cell.

### Immunofluorescence analysis

After HBoV1 infection, ALI membranes were fixed with 3.7% paraformaldehyde in PBS at room temperature for 15 min. The fixed membranes were cut into several small pieces, washed in PBS three times for 5 min, and permeabilized with 0.2% Triton X-100 for 15 min at room temperature. The membranes were then incubated with primary antibody at a dilution of 1∶100 in PBS with 2% FCS for 1 h at 37°C. This was followed by incubation with a fluorescein isothiocyanate- or rhodamine-conjugated secondary antibody. Confocal images were taken with an Eclipse C1 Plus confocal microscope (Nikon, Melville, NY) controlled by Nikon EZ-C1 software. Primary antibodies used were anti-(HBoV1) NS1, NP1 and VP1/2 antibodies, as reported previously [82].

For infected cells cultured in chamber slides, IF analysis was carried out as previously described [83].

### Quantitative PCR (qPCR) analysis

Virus samples were collected from both the apical and basolateral surfaces at multiple time points. Apical washing and harvesting was performed by adding 200 µl of PBS to the apical chamber, incubating the samples for 10 min at 37°C and 5% CO_2_, and removing and storing the 200 µl of PBS from the apical chamber. Thereafter, 50 µl of medium were collected from each basolateral chamber.

Aliquots (100 µl apical or 50 µl basolateral) of the samples were incubated with 25 units of Benzonase (Sigma, St Louis, MO) for 2 h at 37°C, and then digested with 20 µl of proteinase K (15 mg/ml) at 56°C for 10 min. Viral DNA was extracted using QIAamp blood mini kit (Qiagen), and eluted in 100 µl or 50 µl of deionized H_2_O. The extracted DNA was quantified with respect to the number of HBoV1 gc, by a qPCR method that has been used previously [84]. Briefly, the pskHBoV1 plasmid [82], which contains the HBoV1 sequence (nt 1–5299), was used as a control (1 gc = 5.4×10^−12^ µg) to establish a standard curve for absolute quantification with an Applied Biosystems 7500 Fast system (Foster City, CA). The amplicon primers and the PrimeTime dual-labeled probe were designed by Primer Express (version 2.0.0; Applied Biosystems/Life Technologies) and synthesized at IDT Inc. (Coralville, Iowa). Their sequences are as follows (GenBank: JQ411251): forward primers, 5′-GCA CAG CCA CGT GAC GAA-3′ (nt 2391 to 2408); reverse primer, 5′-TGG ACT CCC TTT TCT TTT GTA GGA-3′ (nt 2466 to 2443); and PrimeTime probe, 5′ 6FAM-TGA GCT CAG GGA ATA TGA AAG ACA AGC ATC G-3′ Iowa Black FQ (nt 2411 to 2441). Premix Ex Taq (Takara Bio USA, Madison, WI) was used for qPCR following a standard protocol. 2.5 µl of extracted DNA was used in a reaction volume of 25 µl.

### Histology analysis

On the last day of infection, the HAE on the Millicell inserts were washed with PBS and fixed in 4% paraformaldehyde for ∼30 min. The fixed membranes were cut into several small pieces, and washed with PBS three times. Each membrane fragment was transferred to 20% sucrose in a 15-ml conical tube and allowed to drop to the bottom; it was then embedded vertically in cryoprotectant OCT in an orientation that enabled sectioning of the membrane perpendicular to the blade. Cryostat sections were cut at a thickness of 10 µm, placed onto slides, and stained with hematoxylin and eosin (H&E). Images were taken with a Nikon 80i fluorescence microscope at a magnification of ×60.

## Supporting Information

Figure S1 **Sequencing of PCR DNA fragments.** PCR DNA fragments indicated by arrowheads in Figure 1D were extracted and sequenced. A representative result of sequencing is shown in each chromatogram. The sequences between the arrows in the chromatograms (**A–C**) show the sequences which are complementary to those sequences between the arrows in the hairpin drawings in Figure 1A–C, respectively.
(TIF)

Figure S2 **Construction of a full-length pIHBoV1 clone.** (**A**) **HBoV1 genome.** The full-length genome of HBoV1 is diagramed with structures of the left-end hairpin (LEH) and right-end hairpin (REH) in a form of negative ssDNA from 3′end to 5′end. Restriction enzyme sites of BspEI and BglII in the replicative form (RF) DNA are shown. (**B**) **Cloning of the LEH.** PCR-amplified DNA fragments from the LEH, shown in red, were ligated into pBBSmaI or its derivative. (**C**) **Cloning of the REH.** PCR-amplified or synthesized HBoV1 DNA fragment from the REH, shown in blue, were ligated into pBBSmaI or its derivatives. (**D**) **Cloning of the pIHBoV1.** The pIHBoV1 was constructed by ligating HBoV1 DNA nt 1–517 digested from pBB-LEH and nt 518–4139 amplified from viral DNA extract (HBoV1 Salvador isolate) into the pBB-REH that contains HBoV1 REH (nt 4140–5543). All the numbers are nucleotide numbers of the HBoV1 genome (Genbank JQ923422).
(TIF)

Figure S3 **Kinetics of virus release from HBoV1 infection of primary B31-11 and B29-11 HAE.** Primary B-HAE (donor B31-11 or B29-11) was infected with purified HBoV1 at an MOI of ∼750 genome copy numbers (gc)/cell. Virus was collected from the apical chamber (**A**), or from both the apical and basolateral chambers (**B**) for detection of nuclease-resistant viral gc. Averages and standard deviations are shown. ND, not determined.
(TIF)

Figure S4 **Immunofluorescence analysis of HBoV1-infected HAE polarized from NuLi-1 cells (NuLi-HAE).** NuLi-1 cells were polarized by growth at an ALI for 4 weeks on Millicell inserts of 0.6 cm^2^, until a transepithelial electrical resistance (TEER) of >500 Ω.cm^2^ was detected. Polarized HAE was infected with purified HBoV1 at an MOI of ∼750 gc/cell. At 10 days p.i., infected NuLi-HAE was fixed and stained with an anti-(HBoV1)NS1 antibody. Nuclei were stained with DAPI and cells were visualized by confocal microscopy at a magnification of ×40.
(TIF)

## References

[1] Allander T, Tammi MT, Eriksson M, Bjerkner A, Tiveljung-Lindell A, et al (2005) Cloning of a human parvovirus by molecular screening of respiratory tract samples. Proc Natl Acad Sci U S A 102: 12891–12896. 16118271 10.1073/pnas.0504666102 PMC1200281

[2] Allander T, Jartti T, Gupta S, Niesters HG, Lehtinen P, et al (2007) Human bocavirus and acute wheezing in children. Clin Infect Dis 44: 904–910. 17342639 10.1086/512196 PMC7107819

[3] Schildgen O, Muller A, Allander T, Mackay IM, Volz S, et al (2008) Human bocavirus: passenger or pathogen in acute respiratory tract infections? Clin Microbiol Rev 21: 291–304. 18400798 10.1128/CMR.00030-07 PMC2292574

[4] Kahn J (2008) Human bocavirus: clinical significance and implications. Curr Opin Pediatr 20: 62–66. 18197041 10.1097/MOP.0b013e3282f3f518

[5] Jartti T, Hedman K, Jartti L, Ruuskanen O, Allander T, et al (2011) Human bocavirus-the first 5 years. Rev Med Virol 22: 46–64. 22038931 10.1002/rmv.720

[6] Arthur JL, Higgins GD, Davidson GP, Givney RC, Ratcliff RM (2009) A novel bocavirus associated with acute gastroenteritis in Australian children. PLoS Pathog 5: e1000391. 19381259 10.1371/journal.ppat.1000391 PMC2663820

[7] Kapoor A, Slikas E, Simmonds P, Chieochansin T, Naeem A, et al (2009) A newly identified bocavirus species in human stool. J Infect Dis 199: 196–200. 19072716 10.1086/595831 PMC2678954

[8] Kapoor A, Simmonds P, Slikas E, Li L, Bodhidatta L, et al (2010) Human bocaviruses are highly diverse, dispersed, recombination prone, and prevalent in enteric infections. J Infect Dis 201: 1633–1643. 20415538 10.1086/652416 PMC2902747

[9] Kantola K, Hedman L, Arthur J, Alibeto A, Delwart E, et al (2011) Seroepidemiology of human bocaviruses 1–4. J Infect Dis 204: 1403–1412. 21921203 10.1093/infdis/jir525 PMC3988444

[10] Calvo C, Pozo F, Garcia-Garcia ML, Sanchez M, Lopez-Valero M, et al (2010) Detection of new respiratory viruses in hospitalized infants with bronchiolitis: a three-year prospective study. Acta Paediatr 99: 883–887. 20163373 10.1111/j.1651-2227.2010.01714.x PMC7159545

[11] Garcia-Garcia ML, Calvo C, Falcon A, Pozo F, Perez-Brena P, et al (2010) Role of emerging respiratory viruses in children with severe acute wheezing. Pediatr Pulmonol 45: 585–591. 20503284 10.1002/ppul.21225 PMC7167793

[12] Ursic T, Jevsnik M, Zigon N, Krivec U, Beden AB, et al (2012) Human bocavirus and other respiratory viral infections in a 2-year cohort of hospitalized children. J Med Virol 84: 99–108. 22028039 10.1002/jmv.22217 PMC7167050

[13] Lopez AD, Mathers CD, Ezzati M, Jamison DT, Murray CJ (2006) Global and regional burden of disease and risk factors, 2001: systematic analysis of population health data. Lancet 367: 1747–1757. 16731270 10.1016/S0140-6736(06)68770-9

[14] Shay DK, Holman RC, Newman RD, Liu LL, Stout JW, et al (1999) Bronchiolitis-associated hospitalizations among US children, 1980–1996. JAMA 282: 1440–1446. 10535434 10.1001/jama.282.15.1440

[15] Kantola K, Hedman L, Allander T, Jartti T, Lehtinen P, et al (2008) Serodiagnosis of human bocavirus infection. Clin Infect Dis 46: 540–546. 18199037 10.1086/526532 PMC7107971

[16] Don M, Söderlund-Venermo M, Valent F, Lahtinen A, Hedman L, et al (2010) Serologically verified human bocavirus pneumonia in children. Pediatr Pulmonol 45: 120–126. 19960524 10.1002/ppul.21151

[17] Nascimento-Carvalho CM, Cardoso MR, Meriluoto M, Kemppainen K, Kantola K, et al (2012) Human bocavirus infection diagnosed serologically among children admitted to hospital with community-acquired pneumonia in a tropical region. J Med Virol 84: 253–258. 22170545 10.1002/jmv.22268

[18] Wang K, Wang W, Yan H, Ren P, Zhang J, et al (2010) Correlation between bocavirus infection and humoral response, and co-infection with other respiratory viruses in children with acute respiratory infection. J Clin Virol 47: 148–155. 20022295 10.1016/j.jcv.2009.11.015 PMC7172221

[19] Christensen A, Nordbo SA, Krokstad S, Rognlien AG, Dollner H (2010) Human bocavirus in children: mono-detection, high viral load and viraemia are associated with respiratory tract infection. J Clin Virol 49: 158–162. 20833582 10.1016/j.jcv.2010.07.016 PMC7108378

[20] Söderlund-Venermo M, Lahtinen A, Jartti T, Hedman L, Kemppainen K, et al (2009) Clinical assessment and improved diagnosis of bocavirus-induced wheezing in children, Finland. Emerg Infect Dis 15: 1423–1430. 19788810 10.3201/eid1509.090204 PMC2819894

[21] Ursic T, Steyer A, Kopriva S, Kalan G, Krivec U, et al (2011) Human bocavirus as the cause of a life-threatening infection. J Clin Microbiol 49: 1179–1181. 21227992 10.1128/JCM.02362-10 PMC3067724

[22] Korner RW, Söderlund-Venermo M, van Koningsbruggen-Rietschel S, Kaiser R, Malecki M, et al (2011) Severe human bocavirus infection, Germany. Emerg Infect Dis 17: 2303–2305. 22172367 10.3201/eid1712.110574 PMC3311181

[23] Edner N, Castillo-Rodas P, Falk L, Hedman K, Söderlund-Venermo M, et al (2011) Life-threatening respiratory tract disease with human bocavirus-1 infection in a four-year-old child. J Clin Microbiol 50: 531–532. 22135260 10.1128/JCM.05706-11 PMC3264148

[24] Meriluoto M, Hedman L, Tanner L, Simell V, Makinen M, et al (2012) Association of Human Bocavirus 1 Infection with Respiratory Disease in Childhood Follow-up Study, Finland. Emerg Infect Dis 18: 264–271. 22305021 10.3201/eid1802.111293 PMC3310460

[25] Tijssen P, Agbandje-McKenna M, Almendral JM, Bergoin M, Flegel TW, et al. (2012) Family Parvoviridae. In: King AM, Lefkowitz E, Adams MJ, Carstens EB, editors. Virus Taxonomy: Ninth Report of the International Committee on Taxonomy of Viruses. London: Elsevier. pp. 405–425.

[26] Qiu J, Cheng F, Johnson FB, Pintel D (2007) The transcription profile of the bocavirus bovine parvovirus is unlike those of previously characterized parvoviruses. J Virol 81: 12080–12085. 17715221 10.1128/JVI.00815-07 PMC2168810

[27] Sun Y, Chen AY, Cheng F, Guan W, Johnson FB, et al (2009) Molecular characterization of infectious clones of the minute virus of canines reveals unique features of bocaviruses. J Virol 83: 3956–3967. 19211770 10.1128/JVI.02569-08 PMC2663281

[28] Schildgen O, Qiu J, Söderlund-Venermo M (2012) Genomic features of the human bocaviruses. Future Virol 7: 31–39. 22389649 10.2217/fvl.11.136 PMC3291126

[29] Lusebrink J, Schildgen V, Tillmann RL, Wittleben F, Bohmer A, et al (2011) Detection of head-to-tail DNA sequences of human bocavirus in clinical samples. PLoS ONE 6: e19457. 21573237 10.1371/journal.pone.0019457 PMC3087758

[30] Kapoor A, Hornig M, Asokan A, Williams B, Henriquez JA, et al (2011) Bocavirus episome in infected human tissue contains non-identical termini. PLoS ONE 6: e21362. 21738642 10.1371/journal.pone.0021362 PMC3125170

[31] Dijkman R, Koekkoek SM, Molenkamp R, Schildgen O, van der Hoek L (2009) Human bocavirus can be cultured in differentiated human airway epithelial cells. J Virol 83: 7739–7748. 19474096 10.1128/JVI.00614-09 PMC2708629

[32] Chen KC, Shull BC, Moses EA, Lederman M, Stout ER, et al (1986) Complete nucleotide sequence and genome organization of bovine parvovirus. J Virol 60: 1085–1097. 3783814 10.1128/jvi.60.3.1085-1097.1986 PMC253350

[33] Guan W, Wong S, Zhi N, Qiu J (2009) The genome of human parvovirus B19 virus can replicate in non-permissive cells with the help of adenovirus genes and produces infectious virus. J Virol 83: 9541–9553. 19587029 10.1128/JVI.00702-09 PMC2738243

[34] Luo Y, Chen AY, Qiu J (2011) Bocavirus infection induces a DNA damage response that facilitates viral DNA replication and mediates cell death. J Virol 85: 133–145. 21047968 10.1128/JVI.01534-10 PMC3014208

[35] Wobbe CR, Dean F, Weissbach L, Hurwitz J (1985) In vitro replication of duplex circular DNA containing the simian virus 40 DNA origin site. Proc Natl Acad Sci U S A 82: 5710–5714. 2994044 10.1073/pnas.82.17.5710 PMC390621

[36] Cotmore SF, Tattersall P (2005) A rolling-haipin strategy: basic mechanisms of DNA replication in the parvoviruses. In: Kerr J, Cotmore SF, Bloom ME, Linden RM, Parrish CR, editors. Parvoviruses. London: Hoddler Arond. pp. 171–181.

[37] Cheng F, Chen AY, Best SM, Bloom ME, Pintel D, et al (2009) The capsid proteins of Aleutian mink disease virus (AMDV) activate caspases and are specifically cleaved during infection. J Virol 84: 2687–2696. 20042496 10.1128/JVI.01917-09 PMC2826067

[38] Plevka P, Hafenstein S, Li L, D'Abrgamo A Jr, Cotmore SF, et al (2011) Structure of a packaging-defective mutant of minute virus of mice indicates that the genome is packaged via a pore at a 5-fold axis. J Virol 85: 4822–4827. 21367911 10.1128/JVI.02598-10 PMC3126206

[39] Gonzalez-Mariscal L, Betanzos A, Nava P, Jaramillo BE (2003) Tight junction proteins. Prog Biophys Mol Biol 81: 1–44. 12475568 10.1016/s0079-6107(02)00037-8

[40] Matrosovich MN, Matrosovich TY, Gray T, Roberts NA, Klenk HD (2004) Human and avian influenza viruses target different cell types in cultures of human airway epithelium. Proc Natl Acad Sci U S A 101: 4620–4624. 15070767 10.1073/pnas.0308001101 PMC384796

[41] Villenave R, Thavagnanam S, Sarlang S, Parker J, Douglas I, et al (2012) In vitro modeling of respiratory syncytial virus infection of pediatric bronchial epithelium, the primary target of infection in vivo. Proc Natl Acad Sci U S A 109: 5040–5045. 22411804 10.1073/pnas.1110203109 PMC3323997

[42] Reina J, Ballesteros F, Mari M, Munar M (2001) Evaluation of different continuous cell lines in the isolation of mumps virus by the shell vial method from clinical samples. J Clin Pathol 54: 924–926. 11729211 10.1136/jcp.54.12.924 PMC1731329

[43] Cozens AL, Yezzi MJ, Kunzelmann K, Ohrui T, Chin L, et al (1994) CFTR expression and chloride secretion in polarized immortal human bronchial epithelial cells. Am J Respir Cell Mol Biol 10: 38–47. 7507342 10.1165/ajrcmb.10.1.7507342

[44] Zabner J, Karp P, Seiler M, Phillips SL, Mitchell CJ, et al (2003) Development of cystic fibrosis and noncystic fibrosis airway cell lines. Am J Physiol Lung Cell Mol Physiol 284: L844–L854. 12676769 10.1152/ajplung.00355.2002

[45] Pyrc K, Sims AC, Dijkman R, Jebbink M, Long C, et al (2010) Culturing the unculturable: human coronavirus HKU1 infects, replicates, and produces progeny virions in human ciliated airway epithelial cell cultures. J Virol 84: 11255–11263. 20719951 10.1128/JVI.00947-10 PMC2953148

[46] Cotmore SF, Tattersall P (1987) The autonomously replicating parvoviruses of vertebrates. Adv Virus Res 33:91–174 91–174. 3296697 10.1016/s0065-3527(08)60317-6

[47] Berns KI (1990) Parvovirus replication. Microbiol Rev 54: 316–329. 2215424 10.1128/mr.54.3.316-329.1990 PMC372780

[48] Wang G, Slepushkin VA, Bodner M, Zabner J, van Es HH, et al (1999) Keratinocyte growth factor induced epithelial proliferation facilitates retroviral-mediated gene transfer to distal lung epithelia in vivo. J Gene Med 1: 22–30. 10738582 10.1002/(sici)1521-2254(199901/02)1:1<22::aid-jgm1>3.3.co;2-o

[49] Leigh MW, Kylander JE, Yankaskas JR, Boucher RC (1995) Cell proliferation in bronchial epithelium and submucosal glands of cystic fibrosis patients. Am J Respir Cell Mol Biol 12: 605–612. 7766425 10.1165/ajrcmb.12.6.7766425

[50] Ayers MM, Jeffery PK (1988) Proliferation and differentiation in mammalian airway epithelium. Eur Respir J 1: 58–80. 3284761

[51] Yan Z, Lei-Butters DC, Keiser NW, Engelhardt JF (2012) Distinct transduction difference between adeno-associated virus type 1 and type 6 vectors in human polarized airway epithelia. Gene Ther Jun 14: Epub ahead of print. doi: 10.1038/gt.2012.46. PMID:22695783. 10.1038/gt.2012.46 PMC3443503 22695783

[52] Li W, Zhang L, Johnson JS, Zhijian W, Grieger JC, et al (2009) Generation of novel AAV variants by directed evolution for improved CFTR delivery to human ciliated airway epithelium. Mol Ther 17: 2067–2077. 19603002 10.1038/mt.2009.155 PMC2801879

[53] Limberis MP, Vandenberghe LH, Zhang L, Pickles RJ, Wilson JM (2009) Transduction efficiencies of novel AAV vectors in mouse airway epithelium in vivo and human ciliated airway epithelium in vitro. Mol Ther 17: 294–301. 19066597 10.1038/mt.2008.261 PMC2835069

[54] McCarty DM (2008) Self-complementary AAV vectors; advances and applications. Mol Ther 16: 1648–1656. 18682697 10.1038/mt.2008.171

[55] Excoffon KJ, Koerber JT, Dickey DD, Murtha M, Keshavjee S, et al (2009) Directed evolution of adeno-associated virus to an infectious respiratory virus. Proc Natl Acad Sci U S A 106: 3865–3870. 19237554 10.1073/pnas.0813365106 PMC2646629

[56] Holt PG, Strickland DH, Wikstrom ME, Jahnsen FL (2008) Regulation of immunological homeostasis in the respiratory tract. Nat Rev Immunol 8: 142–152. 18204469 10.1038/nri2236

[57] Fulcher ML, Gabriel S, Burns KA, Yankaskas JR, Randell SH (2005) Well-differentiated human airway epithelial cell cultures. Methods Mol Med 107:183–206 183–206. 15492373 10.1385/1-59259-861-7:183

[58] Karp PH, Moninger TO, Weber SP, Nesselhauf TS, Launspach JL, et al (2002) An in vitro model of differentiated human airway epithelia. Methods for establishing primary cultures. Methods Mol Biol 188: 115–137. 11987537 10.1385/1-59259-185-X:115

[59] Sims AC, Baric RS, Yount B, Burkett SE, Collins PL, et al (2005) Severe acute respiratory syndrome coronavirus infection of human ciliated airway epithelia: role of ciliated cells in viral spread in the conducting airways of the lungs. J Virol 79: 15511–15524. 16306622 10.1128/JVI.79.24.15511-15524.2005 PMC1316022

[60] Zhang L, Peeples ME, Boucher RC, Collins PL, Pickles RJ (2002) Respiratory syncytial virus infection of human airway epithelial cells is polarized, specific to ciliated cells, and without obvious cytopathology. J Virol 76: 5654–5666. 11991994 10.1128/JVI.76.11.5654-5666.2002 PMC137037

[61] Palermo LM, Porotto M, Yokoyama CC, Palmer SG, Mungall BA, et al (2009) Human parainfluenza virus infection of the airway epithelium: viral hemagglutinin-neuraminidase regulates fusion protein activation and modulates infectivity. J Virol 83: 6900–6908. 19386708 10.1128/JVI.00475-09 PMC2698534

[62] Mitchell H, Levin D, Forrest S, Beauchemin CA, Tipper J, et al (2011) Higher level of replication efficiency of 2009 (H1N1) pandemic influenza virus than those of seasonal and avian strains: kinetics from epithelial cell culture and computational modeling. J Virol 85: 1125–1135. 21068247 10.1128/JVI.01722-10 PMC3019989

[63] Widdicombe JH, Widdicombe JG (1995) Regulation of human airway surface liquid. Respir Physiol 99: 3–12. 7740210 10.1016/0034-5687(94)00095-h

[64] Carson JL, Collier AM, Hu SS (1985) Acquired ciliary defects in nasal epithelium of children with acute viral upper respiratory infections. N Engl J Med 312: 463–468. 3969108 10.1056/NEJM198502213120802

[65] Giorgi PL, Oggiano N, Braga PC, Catassi C, Gabrielli O, et al (1992) Cilia in children with recurrent upper respiratory tract infections: ultrastructural observations. Pediatr Pulmonol 14: 201–205. 1484753 10.1002/ppul.1950140402

[66] Tristram DA, Hicks W Jr, Hard R (1998) Respiratory syncytial virus and human bronchial epithelium. Arch Otolaryngol Head Neck Surg 124: 777–783. 9677113 10.1001/archotol.124.7.777

[67] McClure C, Cole KL, Wulff P, Klugmann M, Murray AJ (2011) Production and titering of recombinant adeno-associated viral vectors. J Vis Exp 27: e3348 doi: 10.3791/3348. 10.3791/3348 PMC3308604 22143312

[68] Blessing K, Neske F, Herre U, Kreth HW, Weissbrich B (2009) Prolonged detection of human bocavirus DNA in nasopharyngeal aspirates of children with respiratory tract disease. Pediatr Infect Dis J 28: 1018–1019. 19730155 10.1097/INF.0b013e3181a854ae

[69] Martin ET, Fairchok MP, Kuypers J, Magaret A, Zerr DM, et al (2010) Frequent and prolonged shedding of bocavirus in young children attending daycare. J Infect Dis 201: 1625–1632. 20415535 10.1086/652405 PMC2862123

[70] Brieu N, Guyon G, Rodiere M, Segondy M, Foulongne V (2008) Human bocavirus infection in children with respiratory tract disease. Pediatr Infect Dis J 27: 969–973. 18833027 10.1097/INF.0b013e31817acfaa

[71] Lehtoranta L, Söderlund-Venermo M, Nokso-Koivisto J, Toivola H, Blomgren K, et al (2012) Human bocavirus in the nasopharynx of otitis-prone children. Int J Pediatr Otorhinolaryngol 76: 206–211. 22119148 10.1016/j.ijporl.2011.10.025 PMC7172510

[72] Nakai H, Yant SR, Storm TA, Fuess S, Meuse L, et al (2001) Extrachromosomal recombinant adeno-associated virus vector genomes are primarily responsible for stable liver transduction in vivo. J Virol 75: 6969–6976. 11435577 10.1128/JVI.75.15.6969-6976.2001 PMC114425

[73] Duan D, Sharma P, Yang J, Yue Y, Dudus L, et al (1998) Circular intermediates of recombinant adeno-associated virus have defined structural characteristics responsible for long-term episomal persistence in muscle tissue. J Virol 72: 8568–8577. 9765395 10.1128/jvi.72.11.8568-8577.1998 PMC110267

[74] Norja P, Hedman L, Kantola K, Kemppainen K, Suvilehto J, et al (2012) Occurrence of human bocaviruses and parvovirus 4 in solid tissues. J Med Virol 84: 1276–1273. 10.1002/jmv.23335 22711355

[75] Yan Z, Zak R, Zhang Y, Ding W, Godwin S, et al (2004) Distinct classes of proteasome-modulating agents cooperatively augment recombinant adeno-associated virus type 2 and type 5-mediated transduction from the apical surfaces of human airway epithelia. J Virol 78: 2863–2874. 14990705 10.1128/JVI.78.6.2863-2874.2004 PMC353734

[76] Yan Z, Lei-Butters DC, Liu X, Zhang Y, Zhang L, et al (2006) Unique biologic properties of recombinant AAV1 transduction in polarized human airway epithelia. J Biol Chem 281: 29684–29692. 16899463 10.1074/jbc.M604099200 PMC1712671

[77] Kantola K, Sadeghi M, Antikainen J, Kirveskari J, Delwart E, et al (2010) Real-time quantitative PCR detection of four human bocaviruses. J Clin Microbiol 48: 4044–4050. 20844210 10.1128/JCM.00686-10 PMC3020864

[78] Zhi N, Zadori Z, Brown KE, Tijssen P (2004) Construction and sequencing of an infectious clone of the human parvovirus B19. Virology 318: 142–152. 14972543 10.1016/j.virol.2003.09.011

[79] Qiu J, Pintel DJ (2002) The adeno-associated virus type 2 Rep protein regulates RNA processing via interaction with the transcription template. Mol Cell Biol 22: 3639–3652. 11997501 10.1128/MCB.22.11.3639-3652.2002 PMC133835

[80] Qiu J, Cheng F, Burger LR, Pintel D (2006) The transcription profile of Aleutian Mink Disease Virus (AMDV) in CRFK cells is generated by alternative processing of pre-mRNAs produced from a single promoter. J Virol 80: 654–662. 16378968 10.1128/JVI.80.2.654-662.2006 PMC1346859

[81] Liu Z, Qiu J, Cheng F, Chu Y, Yoto Y, et al (2004) Comparison of the transcription profile of simian parvovirus with that of the human erythrovirus B19 reveals a number of unique features. J Virol 78: 12929–12939. 15542645 10.1128/JVI.78.23.12929-12939.2004 PMC525000

[82] Chen AY, Cheng F, Lou S, Luo Y, Liu Z, et al (2010) Characterization of the gene expression profile of human bocavirus. Virology 403: 145–154. 20457462 10.1016/j.virol.2010.04.014 PMC2879452

[83] Chen AY, Luo Y, Cheng F, Sun Y, Qiu J (2010) Bocavirus infection induces a mitochondrion-mediated apoptosis and cell cycle arrest at G2/M-phase. J Virol 84: 5615–5626. 20335259 10.1128/JVI.02094-09 PMC2876597

[84] Lin F, Zeng A, Yang N, Lin H, Yang E, et al (2007) Quantification of human bocavirus in lower respiratory tract infections in China. Infect Agent Cancer 2: 3. 17266760 10.1186/1750-9378-2-3 PMC1796861

