# Supplementary figures and images for: Establishment of a Reverse Genetics System for Studying Human Bocavirus in Human Airway Epithelia
Source: PLoS Pathog. 2012 Aug 30;8(8):e1002899. doi: 10.1371/journal.ppat.1002899 (PMC3431310; doi:10.1371/journal.ppat.1002899)

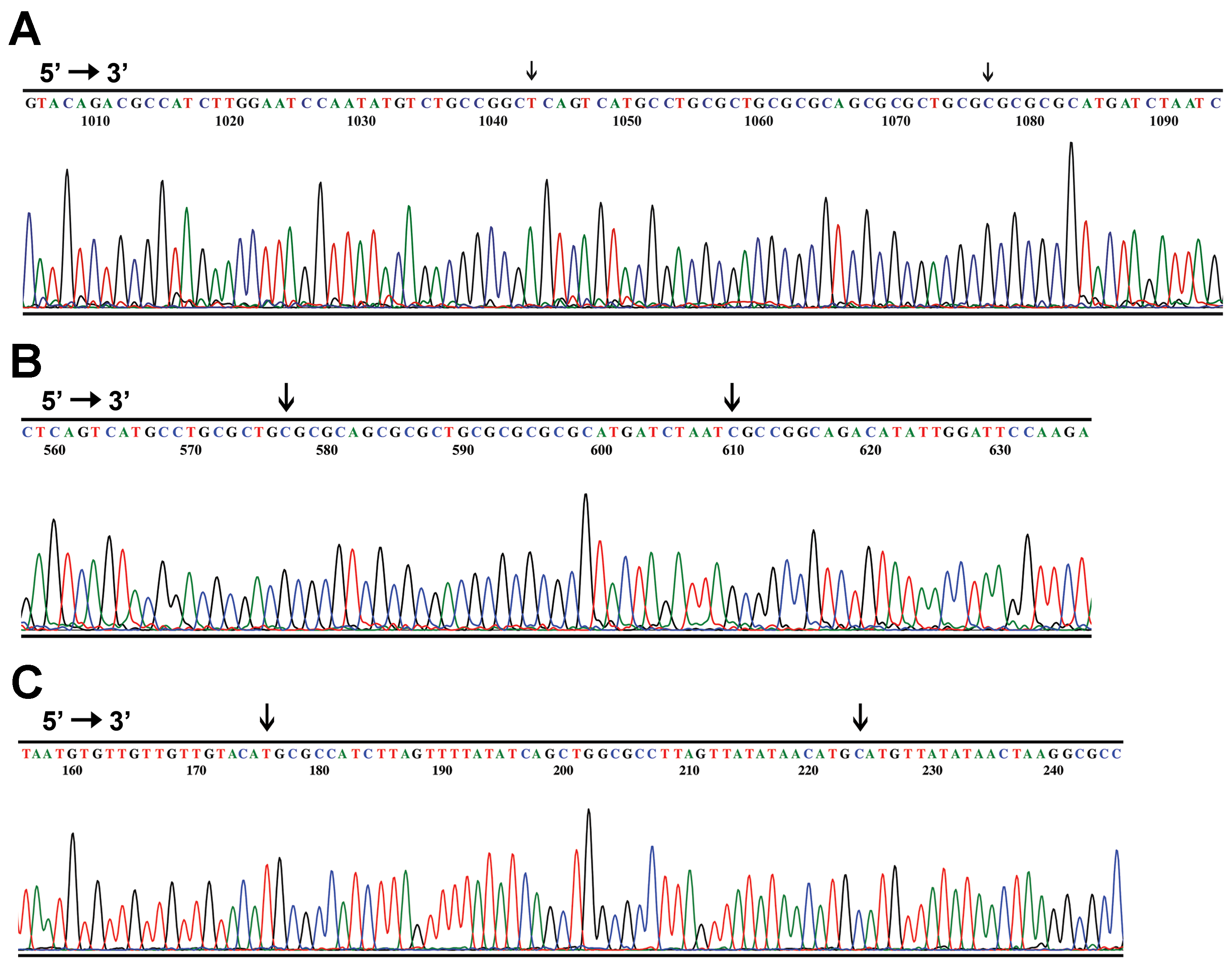

Supplement: Figure S1 — Sequencing of PCR DNA fragments. PCR DNA fragments indicated by arrowheads in Figure 1D were extracted and sequenced. A representative result of sequencing is shown in each chromatogram. The sequences between the arrows in the chromatograms (A–C) show the sequences which are complementary to those sequences between the arrows in the hairpin drawings in Figure 1A–C, respectively. (TIF) [file ppat.1002899.s001.tif]

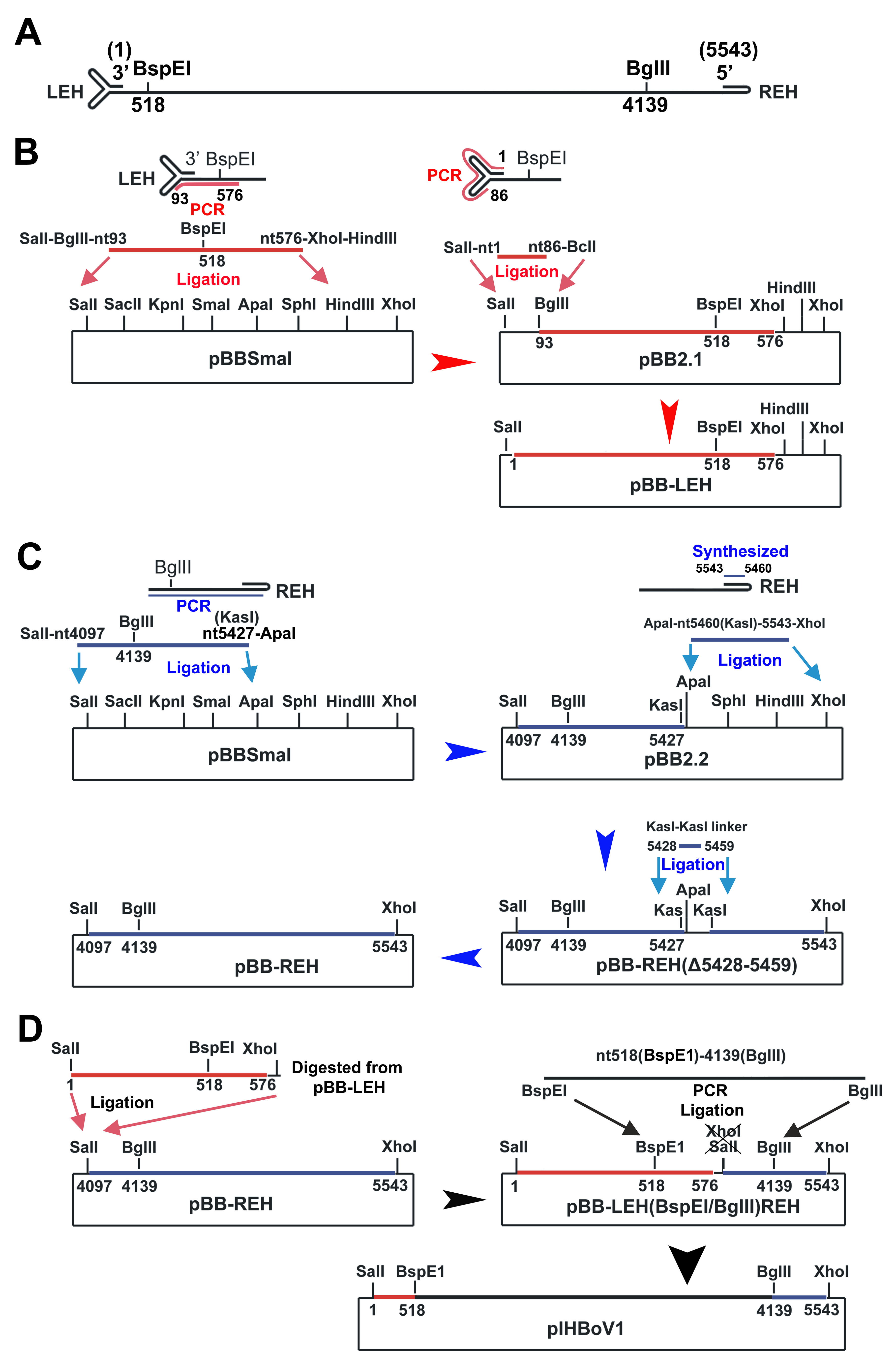

Supplement: Figure S2 — Construction of a full-length pIHBoV1 clone. (A) HBoV1 genome. The full-length genome of HBoV1 is diagramed with structures of the left-end hairpin (LEH) and right-end hairpin (REH) in a form of negative ssDNA from 3′end to 5′end. Restriction enzyme sites of BspEI and BglII in the replicative form (RF) DNA are shown. (B) Cloning of the LEH. PCR-amplified DNA fragments from the LEH, shown in red, were ligated into pBBSmaI or its derivative. (C) Cloning of the REH. PCR-amplified or synthesized HBoV1 DNA fragment from the REH, shown in blue, were ligated into pBBSmaI or its derivatives. (D) Cloning of the pIHBoV1. The pIHBoV1 was constructed by ligating HBoV1 DNA nt 1–517 digested from pBB-LEH and nt 518–4139 amplified from viral DNA extract (HBoV1 Salvador isolate) into the pBB-REH that contains HBoV1 REH (nt 4140–5543). All the numbers are nucleotide numbers of the HBoV1 genome (Genbank JQ923422). (TIF) [file ppat.1002899.s002.tif]

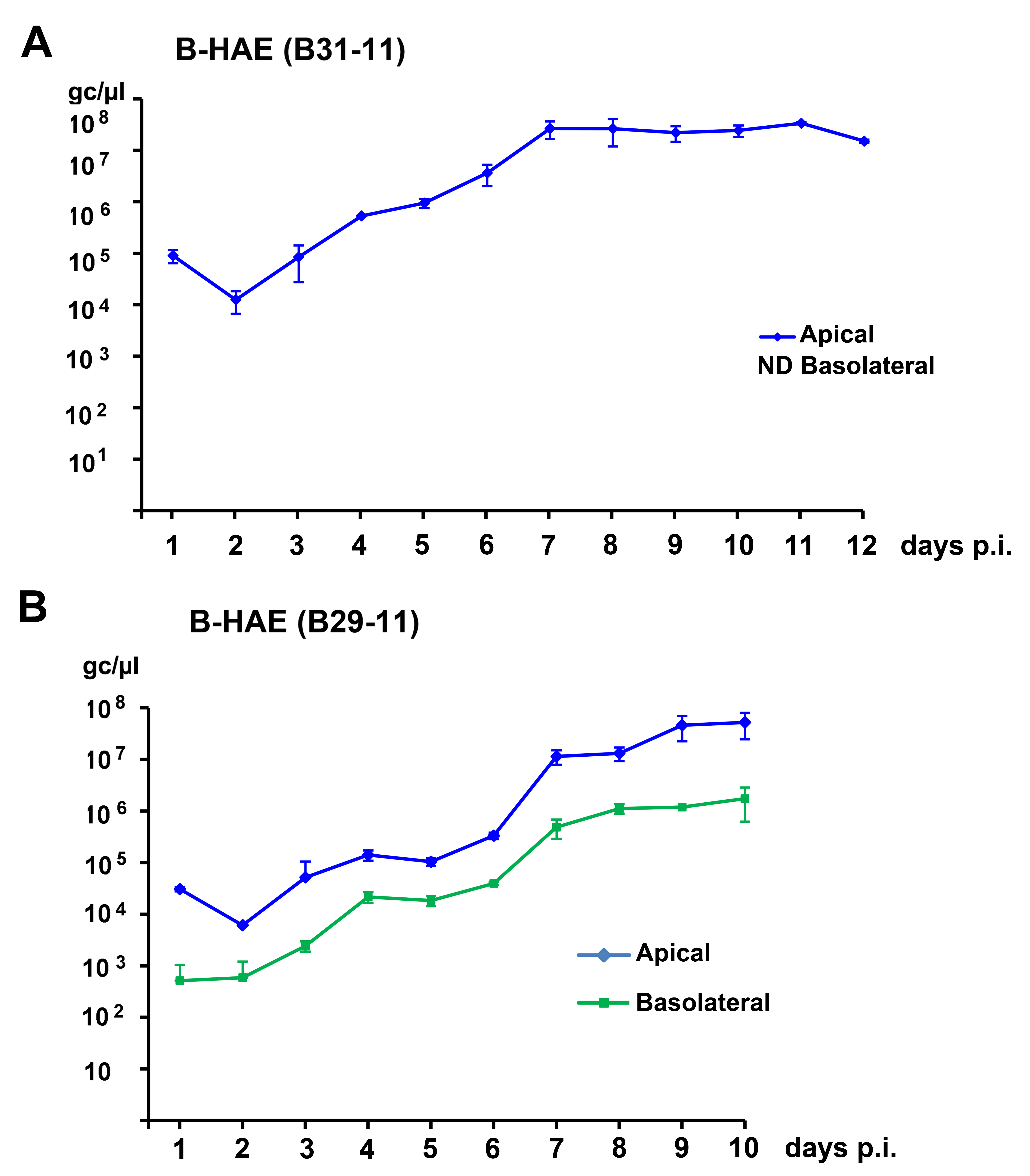

Supplement: Figure S3 — Kinetics of virus release from HBoV1 infection of primary B31-11 and B29-11 HAE. Primary B-HAE (donor B31-11 or B29-11) was infected with purified HBoV1 at an MOI of ∼750 genome copy numbers (gc)/cell. Virus was collected from the apical chamber (A), or from both the apical and basolateral chambers (B) for detection of nuclease-resistant viral gc. Averages and standard deviations are shown. ND, not determined. (TIF) [file ppat.1002899.s003.tif]

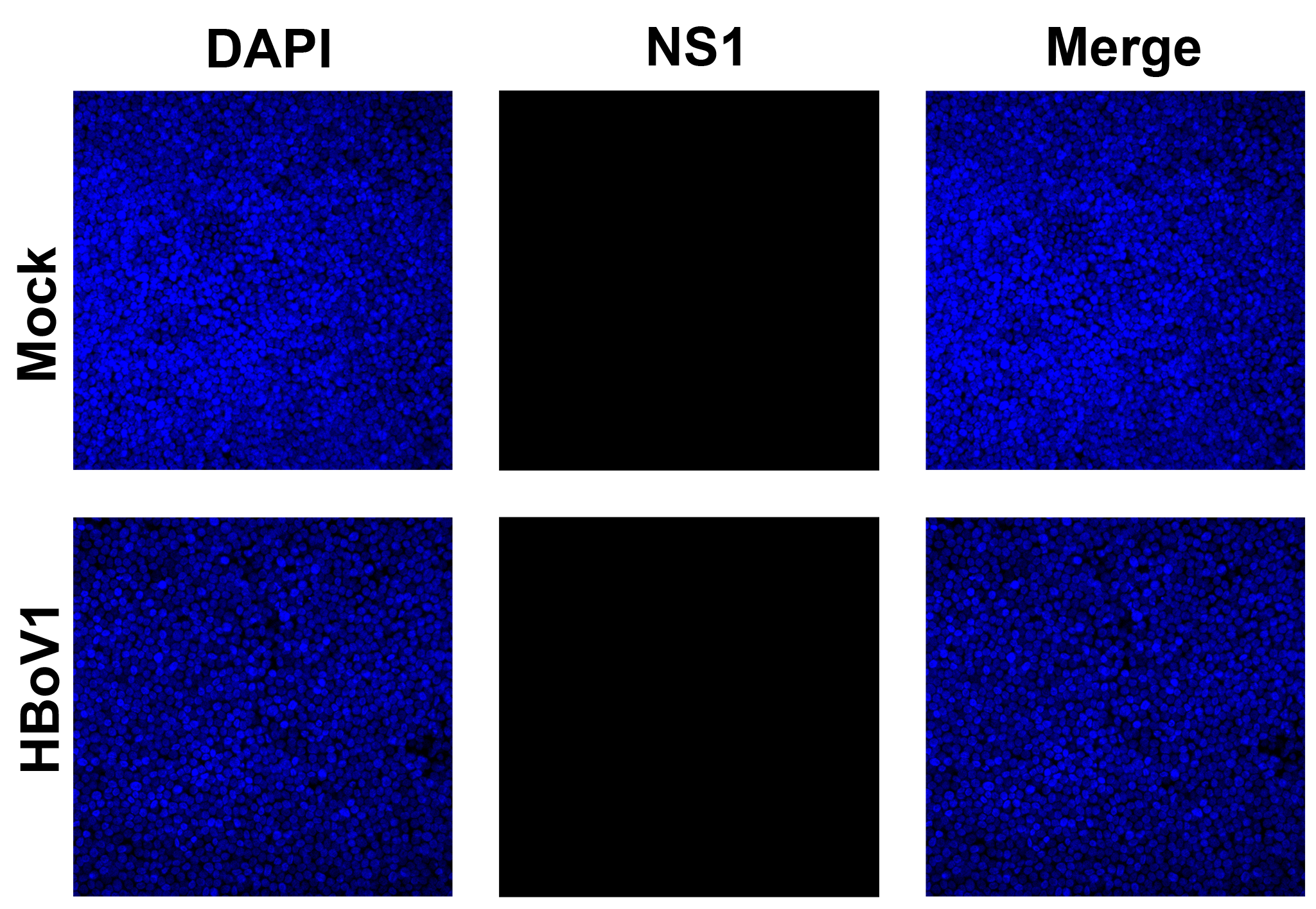

Supplement: Figure S4 — Immunofluorescence analysis of HBoV1-infected HAE polarized from NuLi-1 cells (NuLi-HAE). NuLi-1 cells were polarized by growth at an ALI for 4 weeks on Millicell inserts of 0.6 cm2, until a transepithelial electrical resistance (TEER) of >500 Ω.cm2 was detected. Polarized HAE was infected with purified HBoV1 at an MOI of ∼750 gc/cell. At 10 days p.i., infected NuLi-HAE was fixed and stained with an anti-(HBoV1)NS1 antibody. Nuclei were stained with DAPI and cells were visualized by confocal microscopy at a magnification of ×40. (TIF) [file ppat.1002899.s004.tif]
